# Experimental Study of Capillary-Rise Behavior and Meniscus Evolution in Glass Capillaries Under an Electric Field

**DOI:** 10.3390/mi17070770

**Published:** 2026-06-25

**Authors:** Jiewen Deng, Xingyu Shi, Ning Gu, Guangyuan Kang, Jiacheng Liu

**Affiliations:** 1 School of Energy and Power Engineering, Northeast Electric Power University, Jilin 132012, China; 20182798@neepu.edu.cn (J.D.); 2202400594@neepu.edu.cn (G.K.); 13258788680@163.com (J.L.); 2 Ningxia Zaoquan Electric Power Generation Co., Ltd., Lingwu 750400, China; 18295107622@163.com

**Keywords:** capillary rise, electrocapillary effect, meniscus evolution, electric-field regulation, response surface methodology

## Abstract

To elucidate the scale-dependent response and interfacial evolution of liquid capillary rise in glass capillaries under an electric field, capillaries with different inner diameters were used as model channels. The equilibrium capillary-rise behavior of NaCl solutions without an electric field was investigated, and the coupled effects of capillary diameter, temperature, and concentration were analyzed using response surface methodology. The additional rise of the liquid column under a direct-current electric field was examined, and the interfacial evolution mechanism was explored through meniscus visualization. The results show that, without an electric field, the equilibrium capillary height is governed mainly by capillary inner diameter, followed by temperature, whereas concentration has a relatively weak effect. The developed quadratic regression model shows high fitting accuracy. Under the applied electric field, the electrocapillary response exhibits clear scale selectivity. No significant additional rise was observed in the 0.1 mm and 0.3 mm capillaries, whereas the liquid-column height increased markedly in the 0.5 mm capillary. At 30 °C and 0.75 kV, the additional rise reached 8.2 mm, corresponding to a relative increase of 15.30%. The enhancement at 0.75 kV was stronger than that at 1.5 kV, indicating a non-monotonic voltage response. Meniscus experiments further show that 0.32% NaCl and 5% ethanol solutions respond more evidently to the electric field, with stronger interfacial restructuring for NaCl solution at 0.75 kV. These results indicate that the electric field modifies capillary pressure by altering the force balance near the three-phase contact region and the meniscus curvature, thereby inducing additional liquid-column rise.

## 1. Introduction

The capillary rise of liquids in confined channels is a fundamental process governing mass-transfer efficiency and interfacial stability in porous-media transport, microfluidic chips, bioanalysis, coating wetting, and energy materials [1,2,3,4].

The advancement of microfluidic systems, micro/nanoporous devices and functional interfacial engineering makes controllable liquid transport in microscale confined spaces a critical demand in micromechanics and microsystems. With well-defined boundaries and favorable quantitative tractability, regular cylindrical capillaries are ideal models for characterizing liquid–solid–gas interfacial interactions and elucidating imbibition mechanisms in complex pore networks [5,6], and are widely used to quantify interfacial force evolution and liquid-front migration under external fields [7,8,9,10].

Theoretically, equilibrium capillary-rise height follows Jurin’s law, while the viscosity-dominated transient process is described by the Lucas–Washburn model [11,12,13]; its nanoscale applicability was confirmed by Dimitrov et al. via molecular dynamics simulations [14]. The Young–Lippmann equation, which accounts for electric-field-modulated solid–liquid interfacial free energy and three-phase contact line force balance, serves as the core theoretical framework for electric-field-regulated wettability [15].

However, unlike ideal droplet systems, the continuous meniscus in capillary rise features stronger interfacial constraints and flow coupling, so the classical Young–Lippmann equation cannot fully capture its interfacial displacement and morphological evolution. Electric-field gradients and local force reconstruction near contact lines also markedly affect regulation outcomes [16,17,18,19]. Accordingly, the intrinsic correlation between macroscopic liquid-column rise and microscopic meniscus reconstruction under electric fields remains to be systematically clarified via well-defined experimental systems [20,21,22].

Existing studies confirm electric fields effectively regulate liquid filling and transport in confined channels. For example, Miao and Tsang [23] developed reconfigurability-encoded hierarchical rectifiers for versatile three-dimensional liquid manipulation, demonstrating that structured surfaces combined with external fields can achieve programmable liquid transport. Liu et al. [24] proposed bioinspired capillary transistors based on asymmetric re-entrant structures, enabling programmable control of capillary direction, height, and width. Huh and Mason [25] laid the theoretical foundation for electrically controlled confined flow by eliminating stress singularity at capillary moving contact lines via slip boundary conditions. Siddiqui et al. [26] revealed that electric fields drive flow in partially saturated charged microchannels through coupled electrocapillary and electroosmotic effects, with the dominant mechanism dependent on solution ionic concentration. Electrorheological studies further verified electrocapillary effects modify capillary-filling dynamics, and field-induced rheological changes also affect capillary liquid migration [27].

In applications, Cui et al. [28] utilized electrocapillary effects to accelerate electrolyte wetting in thick dense electrodes for high-energy lithium-ion batteries. Li et al. [29] achieved active reversible switching of capillary flow in conductive nanoporous media by tuning wettability under ultralow voltages. Additionally, electrowetting studies demonstrated that meniscus electric-field response deviates from the linear “higher voltage, stronger wetting” trend, with pronounced nonlinear interfacial reconstruction in extended liquid films and continuous meniscus systems [30,31]. However, most existing studies focus on special fluids, liquid films, or typical electrowetting configurations. Systematic experimental research on the multi-factor coupling and electric-field response of electrolyte capillary rise in conventional glass capillaries remains limited. By contrast, the present work focuses on conventional glass capillaries with sub-millimeter inner diameters. In this intermediate length scale, the electric double layer remains much thinner than the capillary radius, while capillary pressure, hydraulic resistance, contact-line constraints, and electric-field-induced interfacial deformation compete at experimentally observable macroscopic heights.

Three core scientific issues remain unresolved. First, most studies on baseline capillary rise in glass capillaries focus on the individual effects of diameter, temperature, or liquid composition, lacking unified quantitative analysis and statistical modeling of their coupling effects. Second, the scale selectivity of electrocapillary response across capillaries of different sizes remains unclear. Third, the quantitative correlation between macroscopic additional liquid rise and microscopic meniscus curvature evolution is unclarified; apparent contact angle alone cannot fully explain the regulation mechanism, requiring validation via direct meniscus visualization.

In this work, glass capillaries are employed as regular model channels to investigate the electric-field regulation of capillary rise. The zero-field capillary behavior is first characterized as the baseline state. The additional liquid-column rise under a direct-current electric field is then examined for capillaries with different inner diameters. Finally, meniscus visualization and curvature analysis are used to reveal the interfacial mechanism responsible for the observed macroscopic rise. The main novelty of this study lies in identifying the scale-selective electrocapillary response and correlating it with electric-field-induced meniscus reconstruction.

## 2. Experimental Part

### 2.1. Materials and Pretreatment

**Figure 1:**
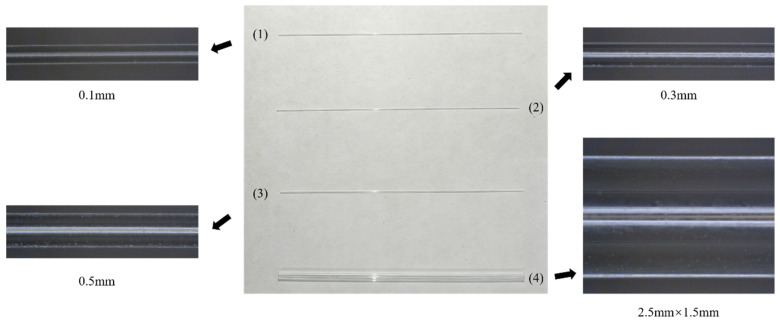
The glass capillary samples used in this study.

Glass capillaries with inner diameters of 0.1, 0.3, and 0.5 mm were used in the capillary-rise and electrocapillary experiments, with an inner-diameter tolerance of ±0.005 mm. For the meniscus-evolution experiments, glass capillaries with an outer diameter of 2.5 mm and an inner diameter of 1.5 mm were employed, with an inner-diameter tolerance of ±0.01 mm. The capillary samples are shown in Figure 1.

Capillaries of different sizes were selected for two different experimental purposes. The capillaries with inner diameters of 0.1, 0.3, and 0.5 mm were used to quantitatively measure capillary-rise height and electric-field-induced additional rise. In contrast, the capillary with an inner diameter of 1.5 mm was used for meniscus visualization because the smaller capillaries did not provide sufficient optical resolution for reliable contour extraction and curvature fitting. Therefore, the meniscus experiments were designed to provide qualitative and semi-quantitative mechanistic evidence of electric-field-induced interfacial deformation, rather than to establish a strict one-to-one quantitative correspondence with the capillary-rise experiments.

All the capillaries were cleaned according to a unified procedure prior to the experiments. First, the capillaries were ultrasonically cleaned in deionized water for 10 min to remove particulate contaminants from the surface. They were then immersed in 0.5 mol/L dilute hydrochloric acid and kept at room temperature for 2 h to remove inorganic residues. After acid treatment, the capillaries were rinsed 3–4 times with deionized water to ensure complete removal of residual hydrochloric acid. Subsequently, they were soaked in ethanol and ultrasonically cleaned for 10 min to remove remaining lipophilic organic compounds. Finally, the capillaries were repeatedly rinsed with deionized water to eliminate any residual chemicals. The cleaned capillaries were dried in a vacuum oven at 105 °C for 2 h and then stored in a clean, dry dish.

The reagents used in the experiments included NaCl, absolute ethanol, glycerol, olive oil, and deionized water. All reagents were of analytical grade and were used without further purification. Their physical properties are listed in Table 1. NaCl solutions were prepared on a mass-fraction basis with target concentrations of 0.06%, 0.32%, and 0.58%. Ethanol–water solution was prepared on a volume-fraction basis with a target ethanol concentration of 5%. The selection of these liquids facilitates the comparative analysis of the contributions of electrical conductivity, polarity, viscosity, and surface tension to electric-field-induced meniscus reconstruction. In the meniscus-evolution experiments, the ratio of outer diameter to inner diameter of the selected capillary was required to be greater than the refractive index of the tested liquid, so as to reduce the boundary distortion caused by refraction and ensure complete imaging of the liquid surface [32].

### 2.2. Experimental Setup

**Figure 2:**
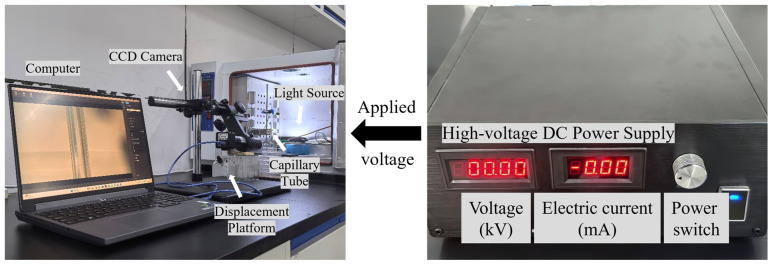
A schematic of the experimental setup for capillary-rise measurement, electric-field loading, and meniscus imaging.

As shown in Figure 2, the capillary-rise and meniscus-evolution experiments shared the same main experimental platform, which mainly consisted of a high-voltage DC power supply, a diffuse light source, a glass capillary, a vertical translation stage, a telecentric lens, an industrial camera, and a computer. The setup was designed to ensure vertical placement of the capillary, controllable liquid-level positioning, stable electric-field loading, and clear acquisition of interfacial images.

For the capillary-rise experiments without an electric field, the capillary was vertically fixed using a polytetrafluoroethylene clamp, which was mounted on the vertical translation stage. The initial immersion depth was precisely adjusted by the stage. The lower end of the capillary was brought into full contact with the liquid without touching the bottom of the dish. The liquid temperature was controlled by a thermostatic water bath.

For the capillary-rise experiments under an electric field, a high-voltage DC power supply, electrodes, and insulation protection components were added to the above setup. The output voltage of the power supply ranged from 0 to 1.5 kV. High-purity copper sheets were used as electrodes, with one placed above the dish and the other immersed in the solution. The vertical distance between the two electrodes was fixed at 40 mm. Therefore, the applied voltages of 0.75 and 1.5 kV corresponded to electric-field strengths of approximately 18.75 and 37.5 kV/m, respectively. The two electrodes were connected to the high-voltage power supply through insulated wires, allowing visual observation and quantitative measurement of both the intrinsic capillary-rise height and the additional rise induced by the electrocapillary effect.

The meniscus-evolution experiments employed the same main structure. An industrial camera and a diffuse backlight source were arranged in the imaging section. The camera was mounted horizontally, with its optical axis perpendicular to the capillary axis, and the liquid–gas interface was positioned within the clear imaging region. A diffuse light source was placed behind the capillary, and the contour evolution of the meniscus under different operating conditions was recorded on a computer for subsequent data processing.

### 2.3. Experimental Procedure

#### 2.3.1. Capillary-Rise Experiments Without an Electric Field

To establish the relationship between liquid rise in regular capillary channels and the subsequent electric-field regulation effect, a single glass capillary was selected as an ideal model system. The effects of capillary inner diameter (0.1, 0.3, and 0.5 mm), NaCl concentration (0.06–0.58%), and experimental temperature (20–40 °C) on the equilibrium capillary height were investigated in the absence of an electric field. The additional rise of the liquid column and the variation in meniscus morphology under an applied electric field were then further analyzed.

During the experiments, the lower end of the capillary was brought into contact with the liquid surface, and the immersion depth was fixed at 2 mm. The liquid-column rise was recorded in real time, and the capillary height was measured after equilibrium had been reached. A new capillary and fresh solution were used for each run. Equilibrium was considered to be reached when the relative variation in liquid-column height did not exceed 0.5% within 5 min.

#### 2.3.2. Capillary-Rise Experiments Under an Applied Electric Field

In each experiment, the equilibrium capillary height at 0 kV was first recorded as the baseline value. The target voltage was then applied, and the height variation of the liquid column was monitored. After the system reached a new stable state, the corresponding capillary height was recorded. A new capillary and fresh solution were used for each experimental run. The electrode plates were reused after standardized cleaning and were replaced if any abnormal condition was observed. After voltage application, the system was regarded as stable when the relative variation in liquid-column height did not exceed 0.5% within 3 min. The absolute resolution of liquid-column height measurement was approximately 0.1 mm, determined by the imaging calibration and scale reading. Therefore, changes smaller than the combined measurement resolution and stability threshold were considered not measurable in the present setup.

To directly observe the interfacial response under an applied electric field, meniscus-evolution experiments were carried out using glass capillaries with a larger inner diameter, and the liquid–gas interface was recorded by a high-resolution industrial camera. In studies related to capillarity, the meniscus is commonly approximated as a spherical cap [22,25,35,36]. Based on this assumption, a circular-arc fitting method was adopted in the present work to extract the meniscus profile.

#### 2.3.3. Meniscus-Evolution Experiments

To improve meniscus visibility, glass capillaries with an outer diameter of 2.5 mm and an inner diameter of 1.5 mm were used. Five liquids were selected as the test media, including absolute ethanol, 5% ethanol solution, olive oil, glycerol, and 0.32% NaCl solution. The experimental temperatures were set at 20, 25, 30, 35, and 40 °C, and the applied voltages were 0, 0.75, and 1.5 kV, resulting in a total of 75 operating conditions. In each run, the steady-state meniscus image at 0 kV was first collected, after which the target voltage was applied and the corresponding image was recorded after the interface had stabilized. A new capillary and fresh test liquid were used for each run. A given condition was considered to have reached steady state when no obvious change in the meniscus was observed in consecutive images and the rate of change in the fitted curvature between adjacent moments was lower than a preset threshold.

### 2.4. Data Processing and Statistical Analysis

The capillary-rise data without an electric field were analyzed using a three-factor, three-level Box–Behnken design (BBD) within the framework of response surface methodology. The three factors were capillary inner diameter (0.1, 0.3, and 0.5 mm), NaCl concentration (0.06%, 0.32%, and 0.58%), and experimental temperature (20, 30, and 40 °C), while the response variable was the equilibrium capillary height. Each factor was assigned three levels, coded as +1, 0, and −1, representing the high, middle, and low levels, respectively. A total of 17 runs were designed, including 12 factorial points and 5 replicated center points. The factors were coded according to Equation (1):

(1) $$x_{i}=\left(X_{i}-X_{0}\right)/\Delta X_{i},$$

where $x_{i}$ is the coded value, $X_{i}$ is the actual value of the independent variable, $X_{0}$ is the actual value at the center point, and $X_{0}$ is the step change.

To describe the relationship between the variables and the response, a quadratic polynomial model was adopted:

(2) $$Y=\beta_{0}+\sum \beta_{i}x_{i}+\sum \beta_{ii}x_{i}^{2}+\sum \beta_{ij}x_{i}x_{j}+\varepsilon ,$$

where Y is the predicted response; $\beta_{0}$ is the constant term; $\beta_{i}$, $\beta_{ii}$, and $\beta_{ij}$ are the regression coefficients of the model; $X_{i}$ and $x_{i}$ are the coded independent variables; and ε represents the random error. Analysis of variance (ANOVA) was used to evaluate the significance and fitting reliability of the regression model.

For the meniscus-evolution experiments, the variation of the meniscus inside the capillary was captured using a camera and a telecentric lens, and the images were acquired using a computer. After image acquisition, the meniscus region was fitted with a circular arc under the spherical-cap approximation. The fitted meniscus curvature was calculated as

(3) $$\kappa =\frac{1}{R}$$

where R is the radius obtained from circular-arc fitting.

To quantify the electric-field-induced curvature variation, the normalized curvature change was defined as

(4) $$\eta_{\kappa }=\frac{\Delta \kappa }{\kappa_{0}}=\frac{\left|\kappa_{U}-\kappa_{0}\right|}{\kappa_{0}}\times 100\%$$

where $\kappa_{0}$ and $\kappa_{0}$ are the fitted meniscus curvatures at 0 kV and under the applied voltage U, respectively.

To compare the capillary-rise experiments and meniscus-visualization experiments conducted at different capillary scales, several dimensionless parameters were evaluated. The relative importance of electric stress to capillary stress was characterized using the electric Bond number,

(5) $$Bo_{e}=\frac{\varepsilon E^{2}R_{c}}{\gamma_{LV}}$$

where ε is the liquid permittivity, E is the nominal electric-field strength, $R_{c}$ is the capillary radius, and $\gamma_{LV}$ is the liquid–vapor surface tension. In addition, the ratio of Debye length to capillary radius was estimated as

(6) $$\frac{\lambda_{D}}{R_{c}}$$

where $\lambda_{D}$ is the Debye length of the electrolyte solution. These dimensionless numbers were used to assess whether the meniscus-visualization experiment can provide mechanistic evidence relevant to the capillary-rise experiments.

## 3. Results and Discussion

### 3.1. Capillary-Rise Behavior in Glass Capillaries Without an Electric Field

The zero-field experiments were conducted to establish a baseline for evaluating the subsequent electric-field-induced modulation. For a cylindrical capillary, the equilibrium capillary height can be described by Jurin’s law:

(7) $$h=\frac{4\gamma_{LV}\cos\theta }{\rho gd},$$

where h is the equilibrium capillary-rise height, θ is the contact angle, ρ is the liquid density, g is the gravitational acceleration, and d is the capillary inner diameter. This equation indicates that, for a given liquid–solid system, the capillary height is mainly governed by the capillary size and the interfacial properties of the liquid.

**Figure 3:**
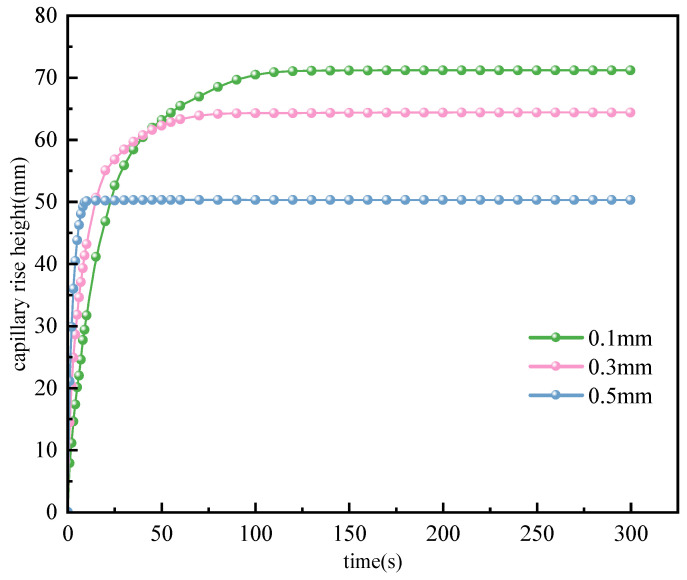
Liquid-column height as a function of time at 30 °C for a 0.32% NaCl solution.

Figure 3 shows a representative time-dependent rise process for the 0.32% NaCl solution at 30 °C. The liquid column rose rapidly at the initial stage and then gradually approached a stable height. This behavior is consistent with the classical capillary-rise process governed by capillary pressure, viscous resistance, and hydrostatic pressure. Therefore, the stable equilibrium height was used as the response variable in the following statistical analysis.

**Figure 4:**
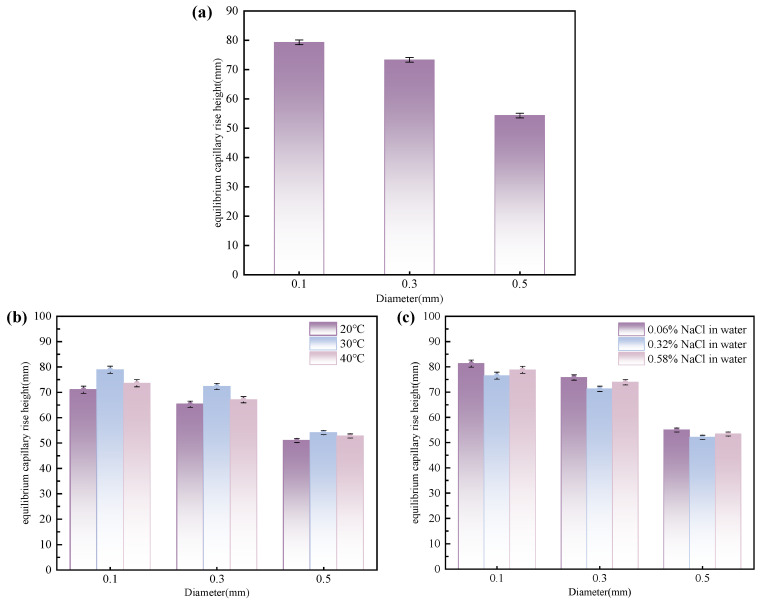
Equilibrium capillary height in glass capillaries without an electric field: (**a**) the effect of capillary inner diameter on the equilibrium height of 0.32% NaCl solution at 30 °C; (**b**) the effect of temperature on the equilibrium height of 0.32% NaCl solution in capillaries with different inner diameters; (**c**) the effect of NaCl concentration on the equilibrium height at 30 °C.

The equilibrium capillary heights in glass capillaries with different inner diameters are shown in Figure 4a. The results show that, under the same liquid composition and environmental conditions, the equilibrium capillary height increased significantly as the capillary inner diameter decreased. This indicates that, in regular capillary channels, geometric scale has a pronounced influence on capillary-rise capacity and is one of the dominant factors controlling the equilibrium capillary height.

This behavior can be explained by the capillary pressure expression. For a cylindrical capillary, when the meniscus is approximated as a spherical cap, the capillary pressure difference can be written as:

(8) $$\Delta P_{c}=\frac{2\gamma_{LV}\cos\theta }{R_{c}},$$

where $\Delta P_{c}$ is the capillary pressure difference.

With stable liquid properties and interfacial wetting, capillary pressure difference increases with decreasing inner diameter, as smaller diameters produce higher meniscus curvature and stronger capillary driving force. This mechanistically explains the dominant role of capillary scale in equilibrium liquid-column height and supports variable weighting in subsequent multivariate statistical analysis. Minor deviations from the ideal model arise from inner-wall roughness, contact angle hysteresis, diameter manufacturing tolerance and measurement uncertainty, necessitating further quantitative evaluation of temperature and concentration effects via response surface methodology (RSM).

Figure 4b shows equilibrium capillary heights of the 0.32% NaCl solution at different temperatures. At a fixed diameter, temperature exerts a weaker, non-monotonic effect, with the maximum height at 30 °C, followed by 40 °C and 20 °C. Per Jurin’s law, temperature indirectly modulates capillary height by altering liquid surface tension, density and wetting state. The competing effects of reduced surface tension and improved glass wettability with rising temperature account for the peak at 30 °C. The temperature-scale coupling effect is further analyzed via the RSM model in the following section.

Figure 4c presents the equilibrium heights of NaCl solutions with varying concentrations at 30 °C. All three solutions exhibit markedly decreasing heights as diameter increases from 0.1 to 0.5 mm, reconfirming that diameter dominates over concentration within the studied range. At a fixed diameter, the 0.06% and 0.58% solutions yield comparable heights, both higher than that of the 0.32% solution. Concentration acts as a modifying rather than controlling factor, affecting height mainly via altering liquid density, surface tension and contact angle without changing the inverse height–diameter trend. This verifies the selected electrolyte concentration provides sufficient conductivity without excessively disturbing baseline capillary rise in subsequent electrocapillary experiments.

Since the above discussion was obtained under the condition that the remaining variables were fixed, response surface methodology was further employed to statistically model the coupled effects of the three factors.

To quantify the relative effects of capillary diameter, temperature, and NaCl concentration, a Box–Behnken response surface design was used. The ANOVA results are summarized in Supplementary Information. The regression model was highly significant, with an F-value of 76.68 and *p* < 0.0001. The coefficient of determination was R^2^ = 0.9900, and the adjusted coefficient was Adj. R^2^ = 0.9770, indicating good fitting reliability. The final fitted quadratic regression model in terms of coded variables was obtained as

(9) $$H=\beta_{0}+\beta_{1}A+\beta_{2}B+\beta_{3}C+\beta_{12}AB+\beta_{13}AC+\beta_{23}BC+\beta_{11}A^{2}+\beta_{22}B^{2}+\beta_{33}C^{2}$$

where *H* is the predicted equilibrium capillary height; *A*, *B*, and *C* represent the coded values of NaCl concentration, temperature, and capillary inner diameter, respectively. Substituting the fitted coefficients gives

(10) $$H=74.1-0.5\times A+2.29\times B-11.66\times C-0.8250\times BC+0.6625\times A^{2}-3.76\times B^{2}-8.76\times C^{2}$$

The coefficient values indicate that the linear term of capillary inner diameter has the largest contribution to the response, followed by the temperature-related terms, whereas the concentration-related terms are comparatively small.

**Figure 5:**
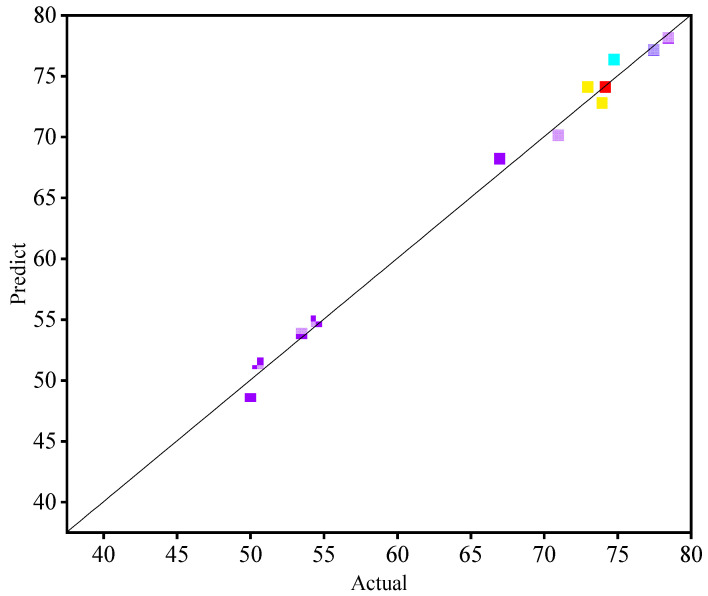
A comparison between the experimental and predicted equilibrium capillary heights.

The comparison between the experimental and predicted capillary heights is shown in Figure 5. The data points are close to the diagonal line, further confirming the reliability of the regression model.

After validating the significance and predictive capability of the regression model, perturbation plots and 3D response surfaces were adopted to visualize factor effects. The perturbation plot compares response sensitivity to each factor at the design center, while 3D surfaces reveal variation trends and pairwise interaction characteristics.

**Figure 6:**
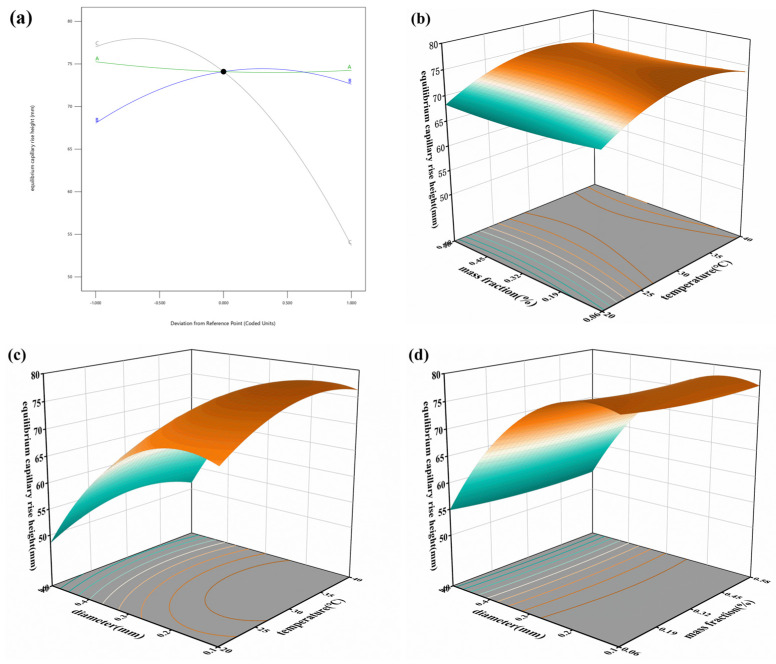
(**a**) A perturbation plot showing the effects of the input variables on the equilibrium capillary height; three-dimensional response surfaces showing the interaction effects between variables: (**b**) x_1_ - x_2_, (**c**) x_2_ - x_3_, and (**d**) x_1_ - x_3_.

As shown in Figure 6a, equilibrium capillary height is most sensitive to capillary inner diameter, followed by temperature, with NaCl concentration exerting the weakest effect. This trend is fully consistent with the single-factor results and ANOVA findings.

Figure 6b indicates that temperature exerts a stronger nonlinear effect on equilibrium height than concentration with weak interaction between the two factors; Figure 6c confirms that capillary inner diameter remains the dominant factor as equilibrium height decreases sharply with increasing diameter, the nonlinear temperature effect is verified, and the temperature-diameter interaction is insignificant; and Figure 6d demonstrates that equilibrium height is still dominated by capillary diameter with concentration only inducing minor fluctuations, and the concentration–diameter interaction is also negligible.

It should be noted that the interaction terms among capillary diameter, temperature, and NaCl concentration were not statistically significant within the investigated parameter range. Therefore, the main role of the RSM analysis in this work is not to reveal strong coupled interactions, but to rank the relative importance of the factors and provide a predictive empirical model for the zero-field baseline.

Overall, the zero-field results are consistent with the physical expectation from Jurin’s law: the equilibrium height decreases with increasing capillary diameter, while temperature and concentration mainly act as secondary modifying factors. Therefore, the zero-field results provide a necessary baseline for interpreting the electric-field-induced additional rise discussed below. Detailed RSM design data are provided in the Supporting Information.

### 3.2. Capillary-Rise Behavior in Glass Capillaries Under an Applied Electric Field

Under zero applied electric field, the rise of the liquid column in a glass capillary is governed by the balance between the capillary pressure difference and the hydrostatic pressure of the liquid column, and the equilibrium capillary height can be described by Jurin’s law. When an external electric field is applied, however, the force balance at the meniscus can be altered through changes in the charge distribution near the interface and in the apparent wetting state. To describe this field-induced wettability variation, the Young–Lippmann relation was introduced here as a qualitative analysis framework:

(11) $$\cos\theta \left(V\right)=\cos\theta_{0}+\frac{\varepsilon_{0}\varepsilon_{r}}{2\gamma_{LV}d}V^{2},$$

where $\theta_{0}$ is the initial effective contact angle in the absence of an electric field, $\theta \left(V\right)$ is the effective contact angle under an applied voltage V, $\varepsilon_{0}$ is the vacuum permittivity, $\varepsilon_{r}$ is the relative permittivity of the equivalent dielectric layer, and d is the thickness of the equivalent dielectric layer.

Although the Young–Lippmann relation is introduced as a reference framework, it should not be interpreted as a strict quantitative model for the present capillary system. In the ideal Young–Lippmann relation, the change in $\Delta \cos\theta \propto U^{2}$. Since Jurin’s law gives h∝cosθ, an ideal electrowetting-type response would predict that the capillary-rise enhancement should increase monotonically with voltage. In particular, increasing the voltage from 0.75 to 1.5 kV would be expected to produce a stronger enhancement under ideal conditions. However, the experimental results show the opposite trend within the tested voltage levels: the additional rise at 0.75 kV was larger than that at 1.5 kV. This discrepancy indicates that the present confined electrolyte capillary system does not follow the ideal Young–Lippmann behavior. The deviation may arise from non-uniform electric fields, interfacial charge redistribution, conductive losses, contact-line constraints, and possible Joule heating. Therefore, the Young–Lippmann equation is used here only to guide the qualitative interpretation of electric-field-induced wetting modification.

For the capillary-rise system considered in this study, the primary role of the electric field is to regulate the interfacial wetting state and thereby affect the meniscus shape, capillary pressure, and final liquid-column height. Therefore, based on the capillary-rise experiments without an electric field, the electric-field variable was introduced to investigate the influence of the applied voltage on capillary response. The applied DC voltage ranged from 0 to 1.5 kV. The working liquid was a 0.32% NaCl solution, and the experimental temperature ranged from 20 to 40 °C.

To quantitatively characterize the additional elevation of the liquid column induced by the electric field, the additional capillary height was defined as:

(12) $$\Delta h=h\left(U\right)-h\left(0\right),$$

where $h\left(U\right)$ is the stable capillary height under the applied voltage U, and $h\left(0\right)$ is the baseline equilibrium capillary height without an electric field. To further compare the relative strength of electric-field enhancement under different capillary sizes and experimental conditions, the relative increase in liquid-column height was defined as:

(13) $$\eta =\frac{h_{U}-h_{0}}{h_{0}}\times 100\%,$$

where η is used to evaluate the relative enhancement induced by the electric field.

#### 3.2.1. Response Characteristics of Liquid-Column Height Under an Applied Voltage

**Figure 7:**
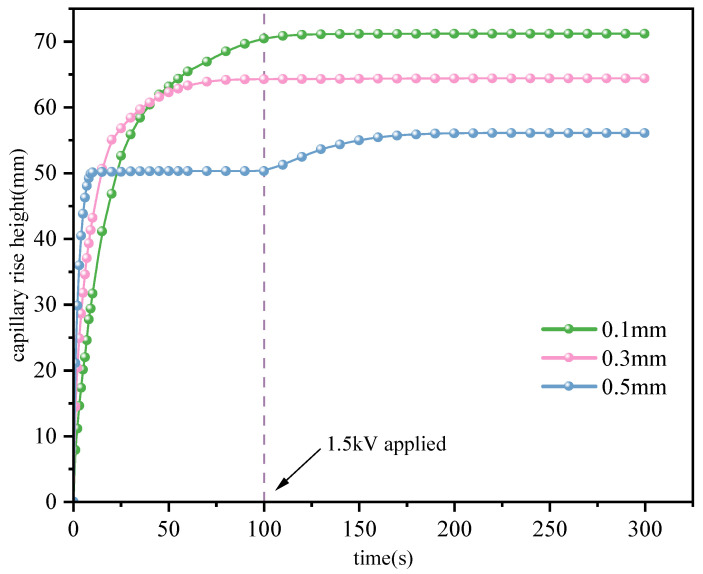
The time evolution of liquid-column height in glass capillaries with different inner diameters under an applied electric field.

Figure 7 shows the time-dependent liquid-column height before and after voltage application in glass capillaries with different inner diameters at 30 °C for a 0.32% NaCl solution. The results indicate that the electric-field response differed significantly with capillary size. For the 0.1 mm and 0.3 mm glass capillaries, once the liquid column reached its equilibrium height under zero electric field, the subsequent application of voltage did not cause any further increase in liquid-column height, and no obvious electrocapillary response was observed. In contrast, for the 0.5 mm glass capillary, when the voltage was applied after the liquid column had reached the equilibrium height without an electric field, the liquid column continued to rise and reached a new stable equilibrium state within a short period.

These results demonstrate that the applied electric field was able to alter the interfacial force balance in the 0.5 mm capillary and induce an additional elevation of the liquid column. More importantly, the effect of the electric field on capillary rise in glass capillaries showed a clear dependence on capillary inner diameter. This difference in dynamic response requires further quantitative evaluation based on the stable capillary height and the additional capillary elevation.

#### 3.2.2. Quantitative Comparison of Electrocapillary Response in Glass Capillaries with Different Inner Diameters

**Figure 8:**
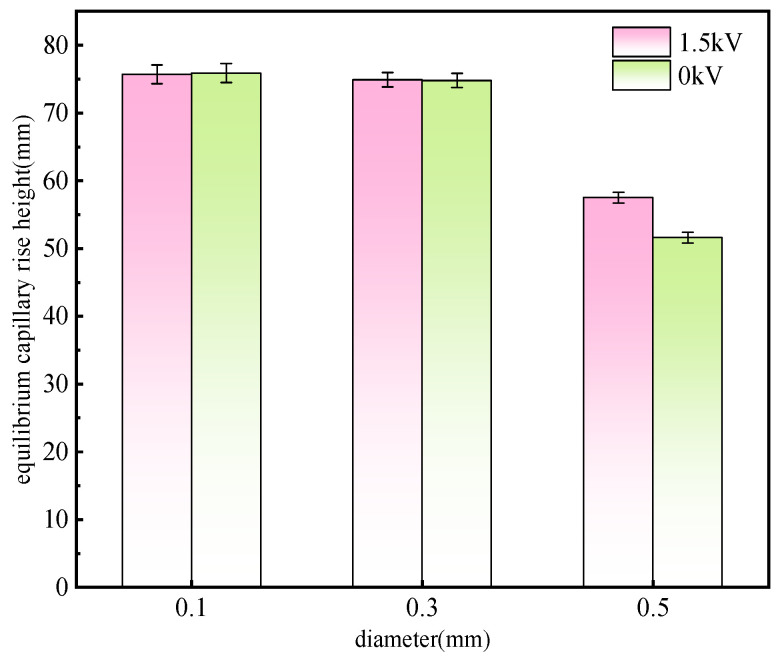
A comparison of equilibrium capillary heights in glass capillaries with different inner diameters under zero electric field and under an applied electric field.

Figure 8 compares the stable capillary-rise heights before and after voltage application for glass capillaries with different inner diameters. As shown in the figure, for the 0.1 mm and 0.3 mm glass capillaries, the difference in stable liquid-column height before and after voltage application was very small, and the two sets of data were essentially comparable within the experimental uncertainty. This indicates that the electric field did not significantly change the equilibrium position of the liquid column in these two smaller capillaries. By contrast, for the 0.5 mm glass capillary, the application of an external electric field of 1.5 kV increased the liquid-column height by 11.43%, indicating that the electric field promoted a further elevation of the liquid column beyond the original capillary rise. This result is consistent with the dynamic response reflected in the liquid-height-versus-time curves in Figure 7.

This behavior is mainly attributed to a mismatch in the scaling of the relevant forces. The additional driving effect generated by the electric field is not sufficiently amplified in narrower capillaries, whereas the viscous resistance increases sharply as the capillary inner diameter decreases. As a result, in the 0.1 mm and 0.3 mm capillaries, the electric-field-induced driving effect was effectively offset by the much larger flow resistance, and no observable additional elevation occurred. Only in the 0.5 mm capillary did the electric-field contribution become sufficiently large to produce a measurable increase in liquid-column height.

When an electric field is applied, the field-induced interfacial contribution can be approximately expressed, in an order-of-magnitude sense, as an additional electric pressure,

(14) $$\Delta p_{e}∼C_{e}E^{2}$$

where E is the effective electric-field strength and $C_{e}$ is an effective coefficient related to the dielectric/electrical properties of the liquid, electrode configuration, and interfacial charge distribution.

Therefore, the relative importance of the electric-field-induced contribution compared with the original capillary pressure can be described by

(15) $$\Pi_{e}=\frac{\Delta p_{e}}{\Delta p_{c}}∼\frac{C_{e}E^{2}d}{4\gamma_{LV}\cos\theta }$$

For a given liquid and applied voltage, $C_{e}$, E, $\gamma_{LV}$, and θ can be regarded as approximately comparable among the three capillaries. Thus, the electric-field contribution relative to the intrinsic capillary pressure scales approximately with the capillary diameter:

(16) $$\Pi_{e}\propto d$$

Taking the 0.5 mm capillary as the reference scale, the diameter ratio is defined as

(17) $$\lambda_{d}=\frac{d}{d_{ref}},d_{ref}=0.5\;\mathrm{mm}$$

Accordingly, the relative electric-field coupling strengths for the 0.1, 0.3, and 0.5 mm capillaries can be estimated as 0.2, 0.6, and 1.0, respectively.

This indicates that the electric-field-induced perturbation is much weaker relative to the intrinsic capillary pressure in the smaller capillaries, whereas it becomes more competitive in the 0.5 mm capillary. In addition, the hydraulic resistance in a cylindrical capillary scales as

(18) $$R_{h}\propto d^{-4}$$

Compared with the 0.5 mm capillary, the hydraulic resistance in the 0.1 and 0.3 mm capillaries is approximately 625 and 7.7 times larger, respectively. Therefore, in the smaller capillaries, the electric-field-induced interfacial perturbation is not only relatively weak compared with the strong baseline capillary pressure but is also more strongly suppressed by viscous resistance and contact-line constraints. This explains why no measurable additional rise was observed in the 0.1 and 0.3 mm capillaries, whereas a clear electrocapillary response appeared in the 0.5 mm capillary.

**Figure 9:**
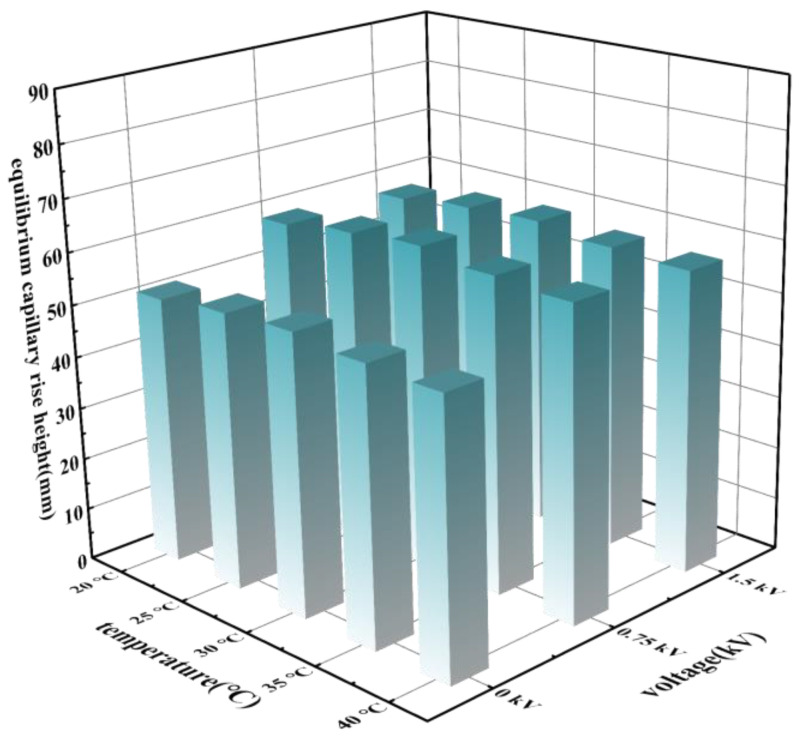
The effects of voltage and temperature on capillary-rise height in the 0.5 mm glass capillary.

Based on the pronounced electrocapillary response observed in the 0.5 mm capillary, the effects of voltage and temperature on the additional capillary elevation were further analyzed. As shown in Figure 9 and Table 2, within the temperature range of 20–40 °C, the applied electric field induced an additional elevation of the liquid column under all investigated conditions. However, the magnitude of this additional elevation did not increase monotonically with increasing voltage. Specifically, at each temperature, the additional capillary height and the relative enhancement ratio at 0.75 kV were higher than those at 1.5 kV. Among all the investigated conditions, the maximum additional capillary height was 8.2 mm and the maximum relative increase reached 15.30% at 30 °C and 0.75 kV. In comparison, although a clear electric-field enhancement effect was still observed at 1.5 kV, the overall magnitude of the additional elevation was slightly smaller than that at 0.75 kV.

These results show that, within the two tested nonzero voltage levels, increasing the voltage from 0.75 to 1.5 kV did not further increase the capillary-rise enhancement. Therefore, the electrocapillary response was not simply proportional to the applied voltage. However, because only two nonzero voltage levels were tested, the complete voltage–response curve cannot be fully determined from the present data. Additional intermediate-voltage measurements, together with conductivity–temperature characterization of the NaCl solution, will be needed in future work to clarify the detailed voltage dependence and the dominant mechanism. The non-monotonic voltage response suggests that the electric-field-induced capillary enhancement is not governed solely by the magnitude of the applied voltage. If the response followed an ideal electrowetting-type relation, a higher voltage would be expected to produce a stronger apparent wetting modification. However, the present capillary system involves an electrolyte solution, a confined meniscus, contact-line constraints, and a non-ideal electric-field distribution. Under these conditions, the effective interfacial response may reach a saturation or enter a nonlinear regime when the voltage is further increased.

The meniscus analysis provides supporting evidence for this interpretation. As discussed in Section 3.3, the fitted meniscus profiles and curvature variations of the 0.32% NaCl solution show a stronger interfacial reconstruction at 0.75 kV than at 1.5 kV under several temperature conditions. This trend is consistent with the larger liquid-column enhancement observed at 0.75 kV. Therefore, the reduced enhancement at 1.5 kV may be associated with a lower effective curvature reconstruction efficiency, possibly caused by interfacial charge redistribution, contact-line stabilization, conductive losses, or local field-induced disturbances. Although the present experiments cannot fully separate these effects, the combined liquid-column and meniscus results indicate that the electrocapillary response is nonlinear within the tested voltage range.

### 3.3. Analysis of Meniscus Evolution and the Mechanism of Electric-Field Regulation

To clarify the mechanism by which an applied electric field affects capillary rise, the morphological response of the meniscus must be analyzed at the liquid–gas interface scale. In a capillary-rise system, the variation in liquid-column height essentially originates from changes in the force balance in the three-phase contact region, which induce an adjustment in meniscus curvature and consequently alter the capillary pressure difference and the final equilibrium height of the liquid column. Therefore, meniscus evolution acts as the key intermediate linking electric-field action to the macroscopic capillary behavior. It should be emphasized that the meniscus-visualization experiments were conducted using a larger capillary with an inner diameter of 1.5 mm, whereas the capillary-rise experiments were performed using capillaries with inner diameters of 0.1, 0.3, and 0.5 mm. This difference in scale was necessary because reliable meniscus contour extraction and curvature fitting require sufficient optical resolution, which could not be achieved in the smaller capillaries. Therefore, the meniscus experiments should not be interpreted as a strict quantitative reproduction of the capillary-rise experiments. Instead, they were used to provide qualitative and semi-quantitative evidence that an applied electric field can reconstruct the meniscus profile and modify the interfacial curvature. To further justify the mechanistic relevance between the two experimental scales, a dimensionless comparison was performed for the 0.32% NaCl solution at 30 °C. The electric Bond number in the 1.5 mm visualization capillary is larger than that in the 0.5 mm capillary because $Bo_{e}\propto R_{c}$ under the same nominal electric-field strength. The Debye length of the 0.32% NaCl solution is on the nanometer scale, and the ratios $\frac{\lambda_{D}}{R_{c}}$ are much smaller than unity for both capillary sizes. Thus, both systems belong to the thin-electric-double-layer limit at the capillary scale. This indicates that the electric-field-induced interfacial deformation is expected to be more readily resolved in the larger capillary, which supports its use for meniscus visualization.

Although the two systems differ in diameter, they are mechanistically connected through the same capillary pressure relation, in which the pressure difference is governed by interfacial curvature and wetting state. The capillary-rise experiments show the macroscopic consequence of electric-field-induced interfacial regulation, whereas the meniscus experiments directly visualize the corresponding interfacial deformation. Thus, the meniscus results are used here to support the proposed mechanism rather than to provide a direct one-to-one quantitative prediction of the liquid-column height.

#### 3.3.1. Basic Morphological Characteristics of Menisci for Different Liquids

**Figure 10:**
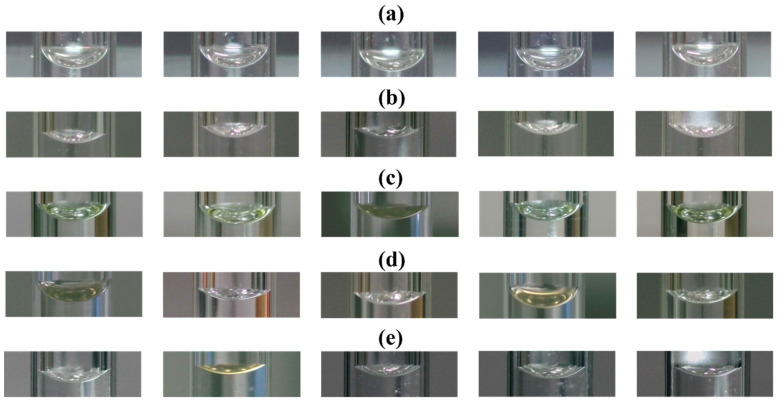
Raw images of menisci for different liquids at different temperatures under 0 kV: (**a**) ethanol, (**b**) 5% ethanol in water, (**c**) olive oil, (**d**) glycerol, and (**e**) 0.32%NaCl in water.

**Figure 11:**
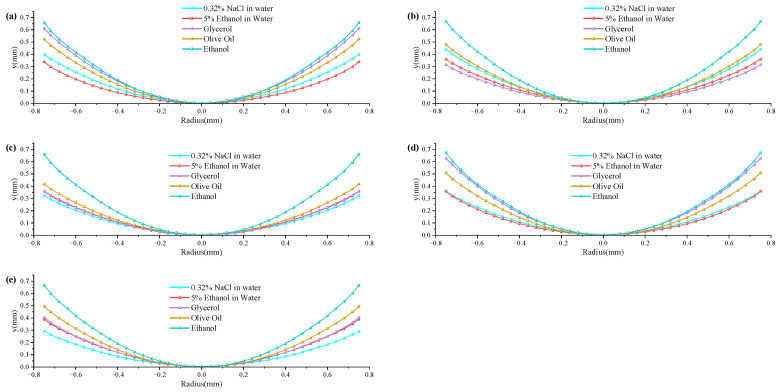
Fitted meniscus profiles for different liquids at different temperatures under 0 kV: (**a**) 20 °C, (**b**) 25 °C, (**c**) 30 °C, (**d**) 35 °C, and (**e**) 40 °C.

Figure 10 presents the raw meniscus images of different liquids under 0 kV, and Figure 11 shows the corresponding fitted profiles. Under the same capillary geometry, clear differences in meniscus shape were observed among the tested liquids, indicating that the initial interfacial equilibrium state was jointly determined by liquid surface tension, density, viscosity, and wetting characteristics on the glass wall.

For capillary-rise processes, meniscus curvature is an important geometric descriptor of capillary pressure. The distinct meniscus shapes formed by different liquids in the absence of an electric field reflect differences in their initial capillary driving force and interfacial wetting state. In addition, temperature variation also caused measurable changes in the meniscus profile, which can be attributed to its combined influence on surface tension, viscosity, density, and contact angle. Therefore, the meniscus morphology under 0 kV can be regarded as the reference state for evaluating electric-field-induced interfacial deformation in the subsequent analysis.

#### 3.3.2. Differences in Meniscus Response of Various Liquids Under an Applied Electric Field

**Figure 12:**
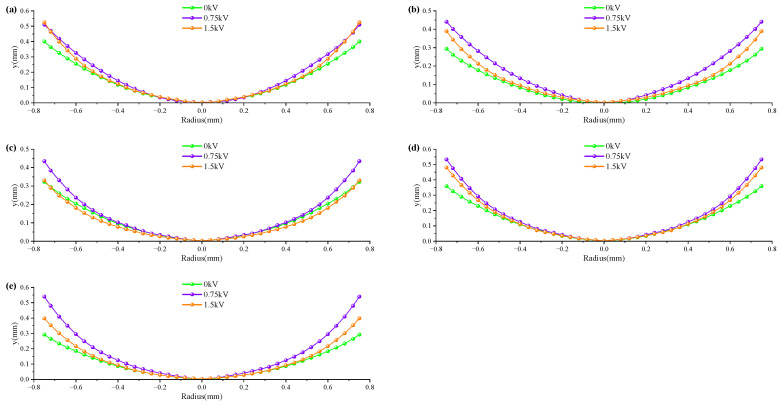
Fitted meniscus profiles of 0.32% NaCl solution under different applied voltages at different temperatures: (**a**) 20 °C, (**b**) 25 °C, (**c**) 30 °C, (**d**) 35 °C, and (**e**) 40 °C.

**Figure 13:**
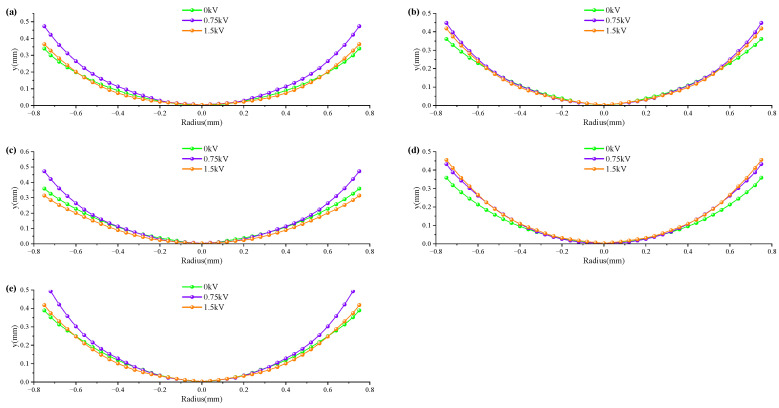
Fitted meniscus profiles of 5% ethanol solution under different applied voltages at different temperatures: (**a**) 20 °C, (**b**) 25 °C, (**c**) 30 °C, (**d**) 35 °C, and (**e**) 40 °C.

Representative fitted profiles of the 0.32% NaCl solution and 5% ethanol solution are shown in Figure 12 and Figure 13. These two liquids exhibited the most evident response to the applied electric field. In contrast, ethanol, glycerol, and olive oil showed only weak or negligible contour variation within the present voltage range, and their full fitting results are provided in the Supporting Information.

These results indicate that the regulation of the meniscus by the applied electric field was strongly liquid-selective, and that the response intensity was closely related to liquid conductivity, polarity, viscosity, and interfacial reconstructability. As an electrolyte solution, the 0.32% NaCl solution has strong ionic conductivity, allowing the electric field to effectively alter the charge distribution and force balance at the liquid–gas interface and in the three-phase contact region, thereby inducing a change in meniscus curvature. The 5% ethanol solution also showed a certain electric-field response because the presence of water increased the polarity of the system and enhanced its coupling with the electric field; in addition, its relatively low viscosity made the interface easier to deform.

By contrast, absolute ethanol had limited electrical conductivity, glycerol exhibited excessively strong viscous damping, and olive oil possessed both weak polarity and low conductivity. Under the present experimental conditions, these three liquids therefore showed no distinguishable meniscus variation. It should be emphasized that this does not necessarily mean that they were completely unaffected by the electric field; rather, under the current voltage range, geometrical scale, and imaging resolution, the interfacial response was insufficient to form a stable and clearly resolvable reconstruction. In essence, this behavior reflects the combined balance among electric-field driving ability, liquid damping, and interfacial coupling efficiency.

Among the two liquids that exhibited obvious electric-field response, the 0.32% NaCl solution was the most relevant to the additional elevation of the liquid column discussed in Section 3.2. As shown in Figure 12, within the temperature range of 20–40 °C, both 0.75 and 1.5 kV significantly altered the interfacial shape of the NaCl solution. Moreover, the meniscus profile shift was more pronounced at 0.75 kV, especially near the wall region. This suggests that, under the present experimental conditions, 0.75 kV induced a stronger regulation of meniscus curvature and a more evident interfacial reconstruction for the NaCl solution, showing the same nonlinear response characteristic as that observed at the macroscopic scale. Figure 13 shows that the 5% ethanol solution also exhibited a certain degree of meniscus-profile displacement under the applied electric field, indicating that this mixed system had some interfacial electro-response capability. However, compared with the NaCl solution, the overall magnitude of meniscus variation was smaller, suggesting that the electric-field/interface coupling in the 5% ethanol solution was weaker than that in the electrolyte solution. This difference is consistent with the differences between the two systems in conductivity, ionic mobility, and interfacial charge-regulation capability.

**Figure 14:**
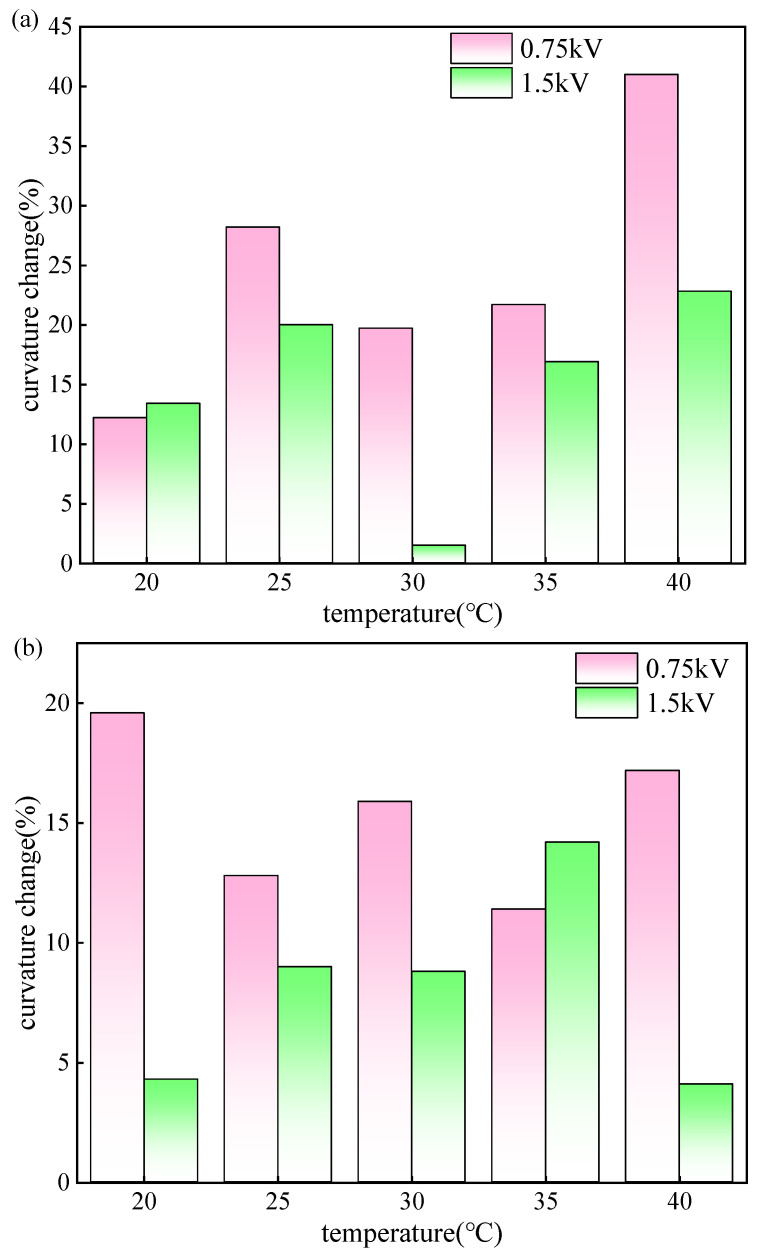
Semi-quantitative results of meniscus curvature changes under applied electric fields: (**a**) 0.32% NaCl, (**b**) 5% ethanol.

The semi-quantitative curvature results are summarized in Figure 14 and Table 3. These values are used to compare relative curvature variations under different electric-field conditions. Because repeated curvature measurements were not available for all conditions, small differences should be interpreted cautiously. Nevertheless, the curvature changes of the 0.32% NaCl solution at 0.75 kV were generally larger than those at 1.5 kV at most temperatures, which is consistent with the larger macroscopic capillary-rise enhancement observed at 0.75 kV. For the 0.32% NaCl solution, the normalized curvature change at 0.75 kV was larger than that at 1.5 kV at most investigated temperatures, except at 20 °C. This trend is consistent with the macroscopic capillary-rise results, where the additional rise at 0.75 kV was larger than that at 1.5 kV. The 5% ethanol solution also exhibited measurable curvature variation, but the magnitude was smaller than that of the NaCl solution. This difference indicates that electrolyte conductivity and interfacial charge regulation play important roles in electric-field-induced meniscus reconstruction.

These observations further indicate that the interfacial regulation by the electric field did not increase monotonically with voltage. For conductive liquid systems, the applied electric field can effectively alter the interfacial charge distribution and apparent wetting state within a certain voltage range, thereby promoting curvature reconstruction of the meniscus. However, when the voltage is further increased, the interfacial response may enter a nonlinear regime or approach saturation. Temperature also modulated the meniscus response under the electric field. Changes in temperature influence the liquid surface tension, viscosity, density, and wetting behavior on the glass wall, and further affect interfacial charge relaxation and electric-field coupling. Therefore, the electrocapillary response should be regarded as the combined result of voltage, temperature, and liquid properties.

## 4. Conclusions

In this work, glass capillaries were used as regular model channels to systematically investigate the capillary-rise behavior without an electric field, the additional elevation of the liquid column under an applied electric field, and the electric-field response characteristics of menisci formed by different liquids. The main conclusions are as follows:

(1) Under zero electric field, the equilibrium capillary height was jointly affected by capillary inner diameter, temperature, and NaCl concentration. Among these factors, capillary inner diameter was the dominant variable, temperature exhibited a nonlinear regulating effect, and the influence of concentration was relatively weak. These trends were further confirmed by the response surface analysis.

(2) The enhancement of capillary rise by the applied electric field showed clear size selectivity. No significant additional elevation was observed in the 0.1 mm and 0.3 mm capillaries, whereas a distinct electrocapillary response was obtained in the 0.5 mm capillary. For the 0.32% NaCl solution, additional liquid-column rise was observed over the temperature range of 20–40 °C, and the enhancement at 0.75 kV was stronger than that at 1.5 kV.

(3) The meniscus-evolution results showed that electric-field regulation was liquid-selective, with obvious interfacial responses observed for the 0.32% NaCl solution and the 5% ethanol solution. Combined with the liquid-column elevation results, it can be concluded that the electric field mainly acted by changing the force balance in the three-phase contact region and reconstructing the meniscus curvature, thereby regulating the capillary pressure difference and inducing additional liquid-column elevation. This process exhibited a nonlinear electric-field response characteristic.

## Figures and Tables

**Table 1:** Physical properties of test liquids [33,34].

Case	Surface Tension Coefficient (γ, mN/m)					Refractive Index (n, 20 °C)	Density ρ, kg/m^3^, 20 °C)
	20 °C	25 °C	30 °C	35 °C	40 °C		
Ethanol	22.3	21.8	21.2	20.7	20.4	1.361	789.300
5% Ethanol in Water	55.9	54.7	53.6	52.5	50.8	1.335	991.100
Olive Oil	32.1	31.83	31.55	31.28	31	1.468	913.000
Glycerol	63.4	62.5	61.6	60.7	59.8	1.475	1263.310
0.32% NaCl in Water	72.97	72.46	71.96	71.47	70.99	1.334	1000.500

**Table 2:** Voltage-induced enhancement of capillary rise at different temperatures.

Temperature	Abs.Enhancement (0.75 kV) (mm)	η (%)	Abs.Enhancement (1.5 kV) (mm)	η (%)
20 °C	7.4	14.42	6.0	11.70
25 °C	7.8	14.77	6.1	11.55
30 °C	8.2	15.30	6.1	11.38
35 °C	7.8	14.89	5.9	11.26
40 °C	7.5	14.45	5.8	11.18

**Table 3:** Semi-quantitative normalized meniscus curvature changes of 0.32% NaCl solution and 5% ethanol solution under different applied voltages.

Liquid	Temperature	$\Delta \kappa /\kappa_{0}$ at 0.75 kV	$\Delta \kappa /\kappa_{0}$ at 1.5 kV	Stronger Response
0.32% NaCl	20 °C	12.2	13.4	1.5 kV
0.32% NaCl	25 °C	28.2	20.0	0.75 kV
0.32% NaCl	30 °C	19.7	1.5	0.75 kV
0.32% NaCl	35 °C	21.7	16.9	0.75 kV
0.32% NaCl	40 °C	41.0	22.8	0.75 kV
5% Ethanol	20 °C	19.6	4.3	0.75 kV
5% Ethanol	25 °C	12.8	9.0	0.75 kV
5% Ethanol	30 °C	15.9	8.8	0.75 kV
5% Ethanol	35 °C	11.4	14.2	1.5 kV
5% Ethanol	40 °C	17.2	4.1	0.75 kV

## Data Availability

The raw data supporting the conclusions of this article will be made available by the authors on request.

## Supplementary Materials

The following supporting information can be downloaded at https://www.mdpi.com/article/10.3390/mi17070770/s1, Figure S1: Raw images of menisci for different liquids at different temperatures under 0.75 kV: (a) ethanol, (b) 5% ethanol in water, (c) olive oil, (d) glycerol, and (e) 0.32%NaCl in water; Figure S2: Raw images of menisci for different liquids at different temperatures under 1.5 kV: (a) ethanol, (b) 5% ethanol in water, (c) olive oil, (d) glycerol, and (e) 0.32%NaCl in water; Figure S3: Fitted meniscus profiles of olive oil under different applied voltages at different temperatures: (a) 20 °C, (b) 25 °C, (c) 30 °C, (d) 35 °C, and (e) 40 °C; Figure S4: Fitted meniscus profiles of glycerol under different applied voltages at different temperatures: (a) 20 °C, (b) 25 °C, (c) 30 °C, (d) 35 °C, and (e) 40 °C; Figure S5: Fitted meniscus profiles of ethanol under different applied voltages at different temperatures: (a) 20 °C, (b) 25 °C, (c) 30 °C, (d) 35 °C, and (e) 40 °C; Table S1: Experimental Design and Results of Response Surface Methodology (RSM); Table S2: ANOVA results of the response surface methodology (RSM) model.

## Abbreviations

The following abbreviations are used in this manuscript:

ANOVA	Analysis of Variance
BBD	Box–Behnken Design
RSM	Response Surface Methodology

## References

[1] Blunt M.J. (2001). Flow in porous media—Pore-network models and multiphase flow. Curr. Opin. Colloid Interface Sci..

[2] Mark D., Haeberle S., Roth G., von Stetten F., Zengerle R. (2010). Microfluidic lab-on-a-chip platforms: Requirements, characteristics and applications. Chem. Soc. Rev..

[3] Dacuycuy S.J., Shiroma W.A., Ohta A.T. (2022). Electrocapillary Actuation of Liquid Metal in Microchannels. Micromachines.

[4] Sydes D., Kler L., Zipfl P., Lutz D., Bouwes H., Huhn C. (2017). On-chip intermediate potential measurements for the control of electromigration in multi-channel networks in case of time-dependent potential changes. Sens. Actuators B Chem..

[5] Shodiev A., Zanotto F.M., Yu J., Chouchane M., Li J., Franco A.A. (2022). Designing electrode architectures to facilitate electrolyte infiltration for lithium-ion batteries. Energy Storage Mater..

[6] Panter J.R., Konicek A.R., King M.A., Squires T.M. (2023). Rough capillary rise. Commun. Phys..

[7] Gelfgat A.Y., Horstmann G.M. (2024). Electrocapillary, thermocapillary, and buoyancy convection driven flows in the Melcher-Taylor experimental setup. Phys. Rev. Fluids.

[8] Wang Y., Zhao Y.-P. (2012). Electrowetting on curved surfaces. Soft Matter.

[9] Kunti G., Bhattacharya A., Chakraborty S. (2017). Numerical investigations of electrothermally actuated moving contact line dynamics. Phys. Fluids.

[10] Orejon D., Sefiane K., Shanahan M.E.R. (2013). Young-Lippmann equation revisited for nanosuspensions. Appl. Phys. Lett..

[11] Jurin J. (1718). An account of some observations concerning the ascent of water in small glass tubes. Philos. Trans. R. Soc..

[12] Washburn E.W. (1921). The dynamics of capillary flow. Phys. Rev..

[13] Zhmud B.V., Tiberg F., Hallstensson K. (2000). Dynamics of capillary rise. J. Colloid Interface Sci..

[14] Dimitrov D.I., Milchev A., Binder K. (2007). Capillary rise in nanopores: Molecular dynamics evidence for the Lucas-Washburn equation. Phys. Rev. Lett..

[15] Lee C.P., Fang B.Y., Wei Z.H. (2013). Influence of electrolytes on contact angles of droplets under electric field. Analyst.

[16] Wang W., Wang Q., Zhou J., Riaud A. (2021). Observation of contact angle hysteresis due to inhomogeneous electric fields. Commun. Phys..

[17] Johansson P., Hess B. (2020). Electrowetting diminishes contact line friction in molecular wetting. Phys. Rev. Fluids.

[18] Dhar J., Chakraborty S. (2018). Electrically modulated capillary filling imbibition of nematic liquid crystals. Phys. Rev. E.

[19] Ye J., Tan S.-C., Wang L., Liu J. (2021). A new hydrodynamic interpretation of liquid metal droplet motion induced by an electrocapillary phenomenon. Soft Matter.

[20] Kim H., Lim J.H., Lee K., Choi S.Q. (2020). Direct measurement of contact angle change in capillary rise. Langmuir.

[21] Siebold A., Nardin M., Schultz J., Walliser A., Oppliger M. (2000). Effect of dynamic contact angle on capillary rise phenomena. Colloids Surf. A.

[22] Xue Y.H., Markmann J., Duan H.L., Weissmüller J., Huber P. (2014). Switchable imbibition in nanoporous gold. Nat. Commun..

[23] Miao J.Q., Tsang A.C.H. (2024). Reconfigurability-Encoded Hierarchical Rectifiers for Versatile 3D Liquid Manipulation. Adv. Sci..

[24] Liu X., Gao M., Li B., Liu R., Chong Z., Gu Z., Zhou K. (2024). Bioinspired Capillary Transistors. Adv. Mater..

[25] Huh C., Mason S.G. (1977). The steady movement of a liquid meniscus in a capillary tube. J. Fluid Mech..

[26] Siddiqui M.A.Q., Sadeghinezhad E., Regenauer-Lieb K., Roshan H. (2022). Electrolytic flow in partially saturated charged micro-channels: Electrocapillarity versus electroosmosis. Phys. Fluids.

[27] Melcher J.R., Taylor G.I. (1969). Electrodynamics: Interfacial shear stresses. Annu. Rev. Fluid Mech..

[28] Cui H., Song Y.Z., Ren D.S., Wang L., He X. (2024). Electrocapillary boosting electrode wetting for high-energy lithium-ion batteries. Joule.

[29] Li X.Y., Xue Y.H., Duan H.L. (2017). Electrocapillary rise in nanoporous media. Procedia IUTAM.

[30] Quilliet C., Berge B. (2001). Electrowetting: A recent outbreak. Curr. Opin. Colloid Interface Sci..

[31] Saville D.A. (1997). Electrodynamics: The Taylor-Melcher leaky dielectric model. Annu. Rev. Fluid Mech..

[32] Han G., Li K.Y., Yang Y.Y., Yu B., Zhou H. (2025). High precision measurement of meniscus profile on liquid column in cylindrical capillary based on telecentric imaging technology. Opt. Express.

[33] Horibe A., Fukusako S., Yamada M. (1996). Surface Tension of Low-Temperature Aqueous Solutions. Int. J. Thermophys..

[34] Xu T., Rodriguez-Martinez V., Sahasrabudhe S.N., Farkas B.E., Dungan S.R. (2017). Effects of Temperature, Time and Composition on Food Oil Surface Tension. Food Biophys..

[35] Chatterjee J. (2007). Prediction of coupled menisci shapes by Young-Laplace equation and the resultant variability in capillary retention. J. Colloid Interface Sci..

[36] de Gennes P.G., Quéré D., Brochard-Wyart F. (2004). Capillarity and Wetting Phenomena: Drops, Bubbles, Pearls, Waves.

