# Supplementary material for: Experimental Study of Capillary-Rise Behavior and Meniscus Evolution in Glass Capillaries Under an Electric Field
Source: Micromachines (Basel). 2026 Jun 25;17(7):770. doi: 10.3390/mi17070770 (PMC13413913; doi:10.3390/mi17070770)
Supplement: Supplementary file 1 [file micromachines-17-00770-s001.zip › Table S2.pdf]

Table S2 ANOVA results of the response surface methodology (RSM) model.

Source	Sum of squares	Degree of freedom	Mean square	F-value	p-value	
Model	1533.31	9	170.37	76.68	<0.0001	significant
x_1-mass fraction	2.00	1	2.00	0.9002	0.3743	
x_2-temperature	41.86	1	41.86	18.84	0.0034	
x_3-diameter	1088.11	1	1088.11	489.75	<0.0001	
x_1x_2	0.0000	1	0.0000	0.0000	1.0000	
x_1x_3	0.0000	1	0.0000	0.0000	1.0000	
x_2x_3	2.72	1	2.72	1.23	0.3049	
x_1^2	1.85	1	1.85	0.8318	0.3921	
x_2^2	59.61	1	59.61	26.83	0.0013	
x_3^2	323.29	1	323.29	145.51	<0.0001	
Residual	15.55	7	2.22			
Lack of Fit	12.67	3	4.22	5.87	0.0602	not significant
Pure Error	2.88	4	0.7200			
Cor Total	1548.86	16				
