# Supplementary material for: Experimental Study of Capillary-Rise Behavior and Meniscus Evolution in Glass Capillaries Under an Electric Field
Source: Micromachines (Basel). 2026 Jun 25;17(7):770. doi: 10.3390/mi17070770 (PMC13413913; doi:10.3390/mi17070770)
Supplement: Supplementary file 1 [file micromachines-17-00770-s001.zip › Table S1.pdf]

Table S1. Experimental Design and Results of Response Surface Methodology (RSM).

| Run | A：Mass fraction(%) | | B：Temperature(℃) | | C：Diameter(mm) | | Capillary rise height（mm） |
|---|---|---|---|---|---|---|---|
| | $x_1$ | $X_1$ | $x_2$ | $X_2$ | $x_3$ | $X_3$ | |
| 1 | 0 | 0.32 | -1 | 20 | +1 | 0.5 | 50 |
| 2 | -1 | 0.06 | -1 | 20 | 0 | 0.3 | 68 |
| 3 | +1 | 0.58 | -1 | 20 | 0 | 0.3 | 67 |
| 4 | 0 | 0.32 | 0 | 30 | 0 | 0.3 | 73 |
| 5 | 0 | 0.32 | +1 | 40 | -1 | 0.1 | 74.8 |
| 6 | 0 | 0.32 | -1 | 20 | -1 | 0.1 | 71 |
| 7 | +1 | 0.58 | 0 | 30 | +1 | 0.5 | 53.5 |
| 8 | 0 | 0.32 | 0 | 30 | 0 | 0.3 | 74.8 |
| 9 | -1 | 0.06 | 0 | 30 | -1 | 0.1 | 78.5 |
| 10 | 0 | 0.32 | +1 | 40 | +1 | 0.5 | 50.5 |
| 11 | +1 | 0.58 | +1 | 40 | 0 | 0.3 | 74 |
| 12 | 0 | 0.32 | 0 | 30 | 0 | 0.3 | 73.5 |
| 13 | 0 | 0.32 | 0 | 30 | 0 | 0.3 | 75 |
| 14 | 0 | 0.32 | 0 | 30 | 0 | 0.3 | 74.2 |
| 15 | -1 | 0.06 | +1 | 40 | 0 | 0.3 | 75 |
| 16 | -1 | 0.06 | 0 | 30 | +1 | 0.5 | 54.5 |
| 17 | +1 | 0.58 | 0 | 30 | -1 | 0.1 | 77.5 |
