# Supplementary figures and images for: Experimental Study of Capillary-Rise Behavior and Meniscus Evolution in Glass Capillaries Under an Electric Field
Source: Micromachines (Basel). 2026 Jun 25;17(7):770. doi: 10.3390/mi17070770 (PMC13413913; doi:10.3390/mi17070770)

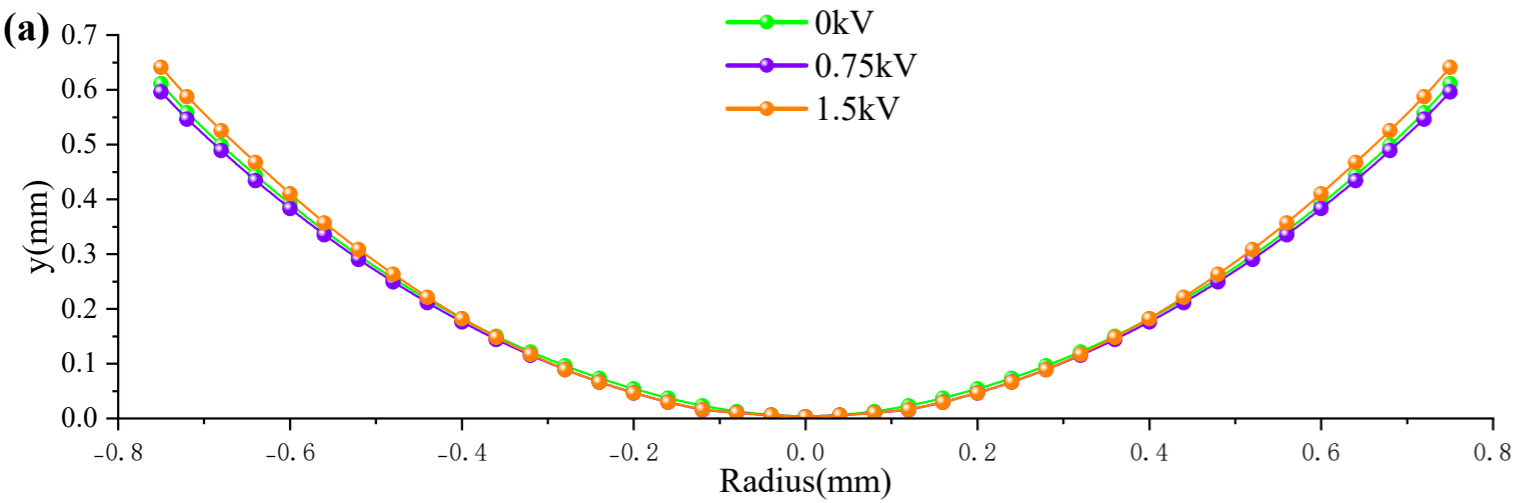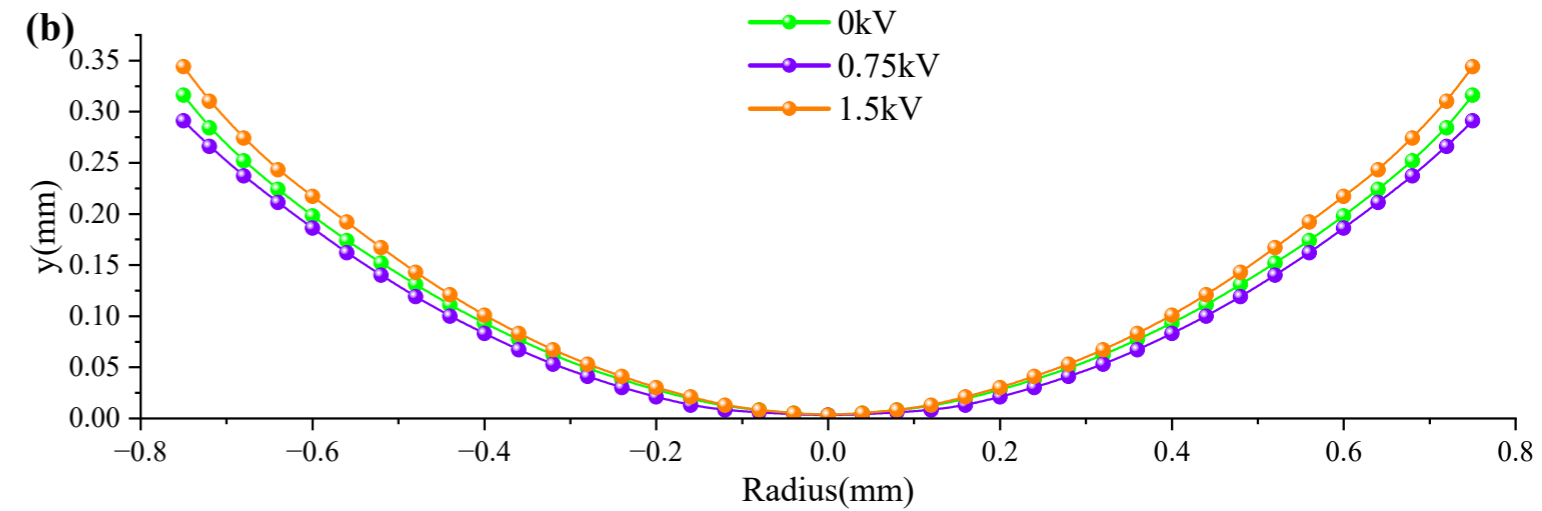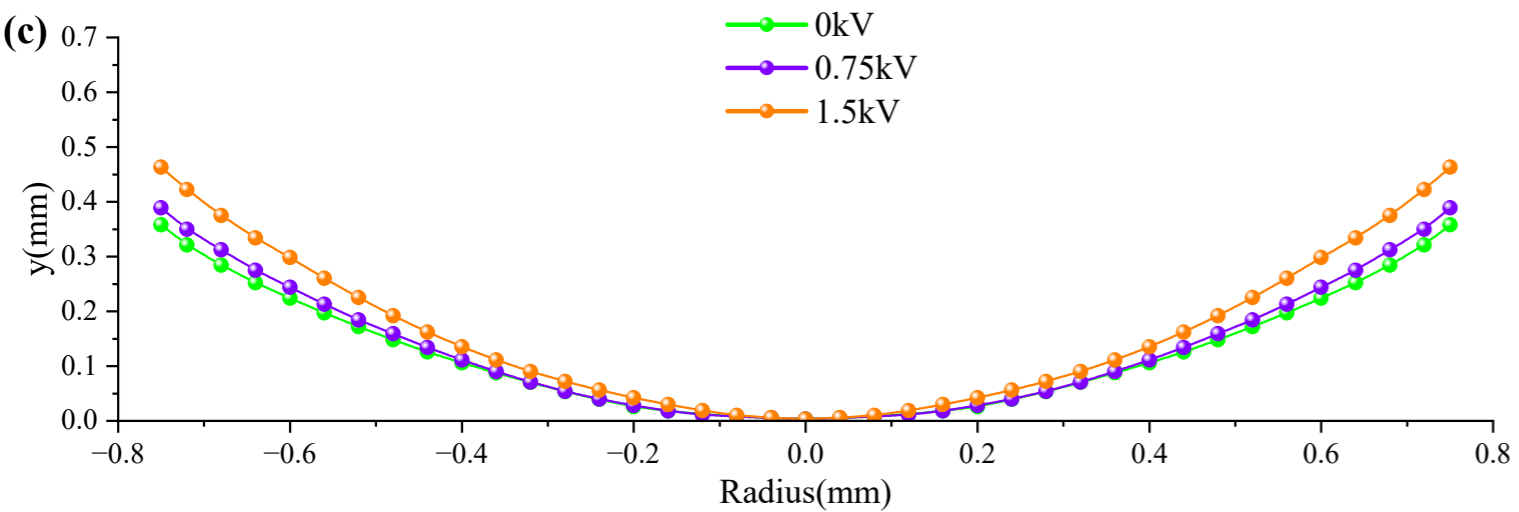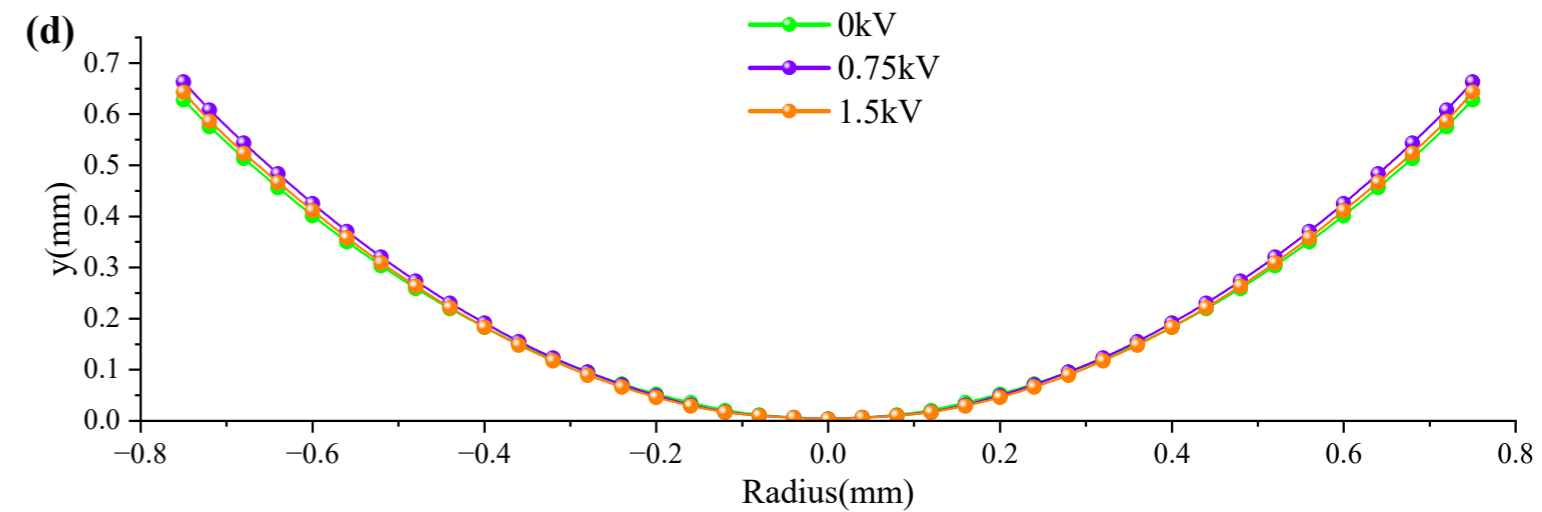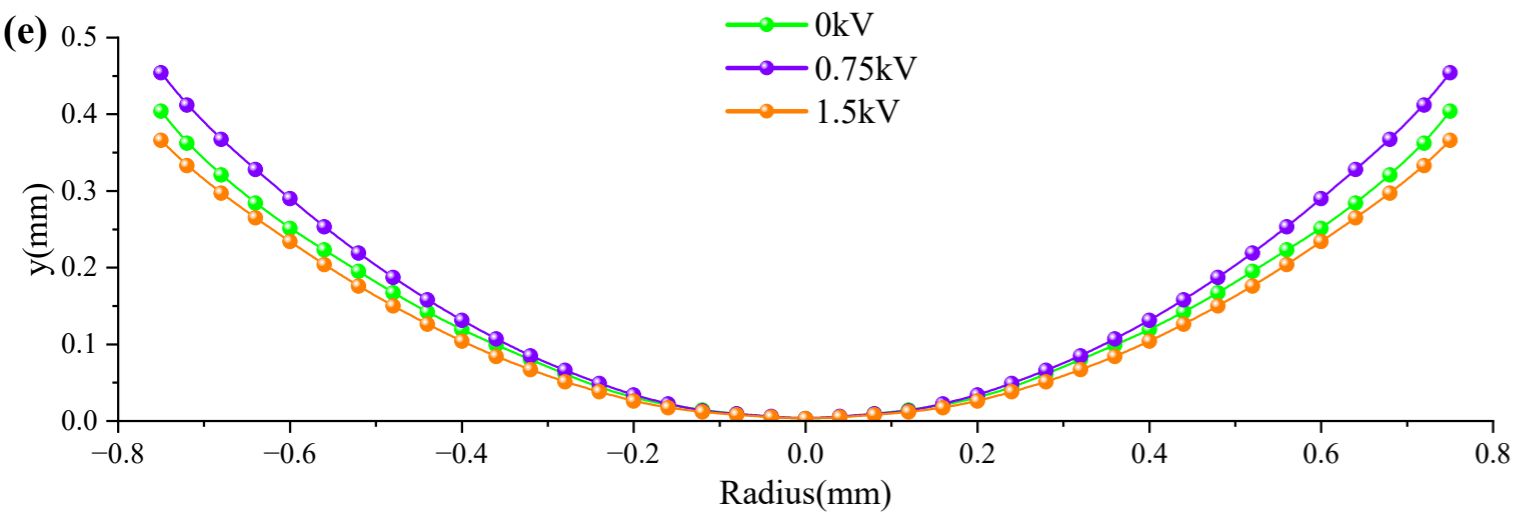

Supplement: Supplementary file 1 [file micromachines-17-00770-s001.zip › figS4.pdf]

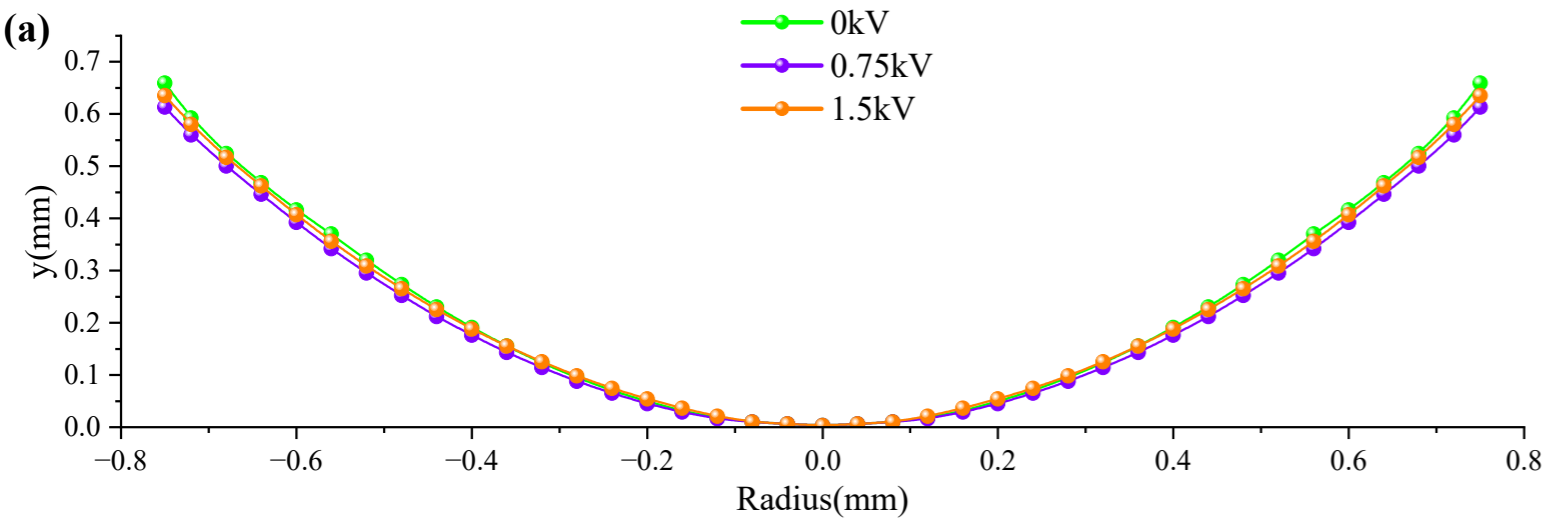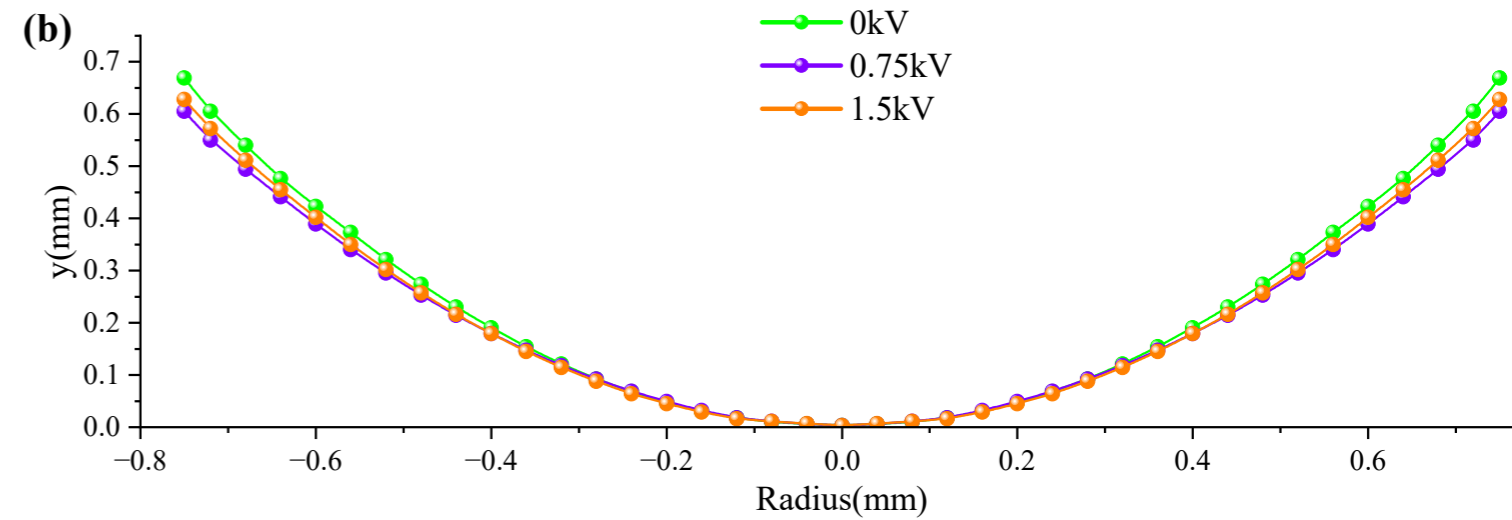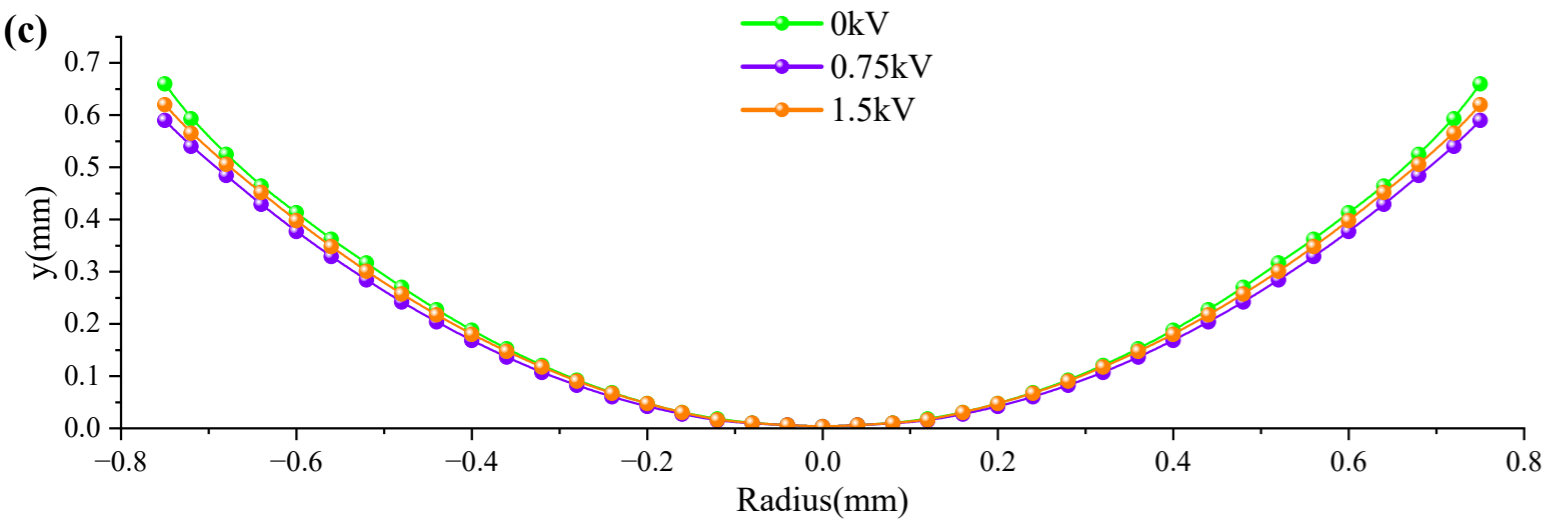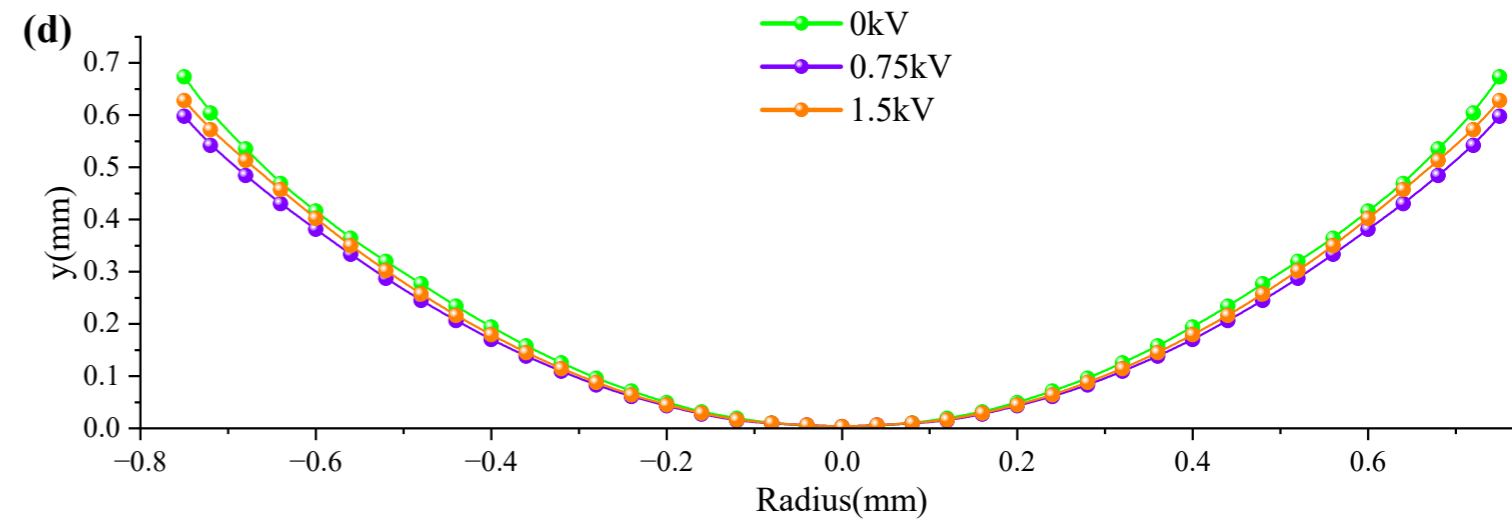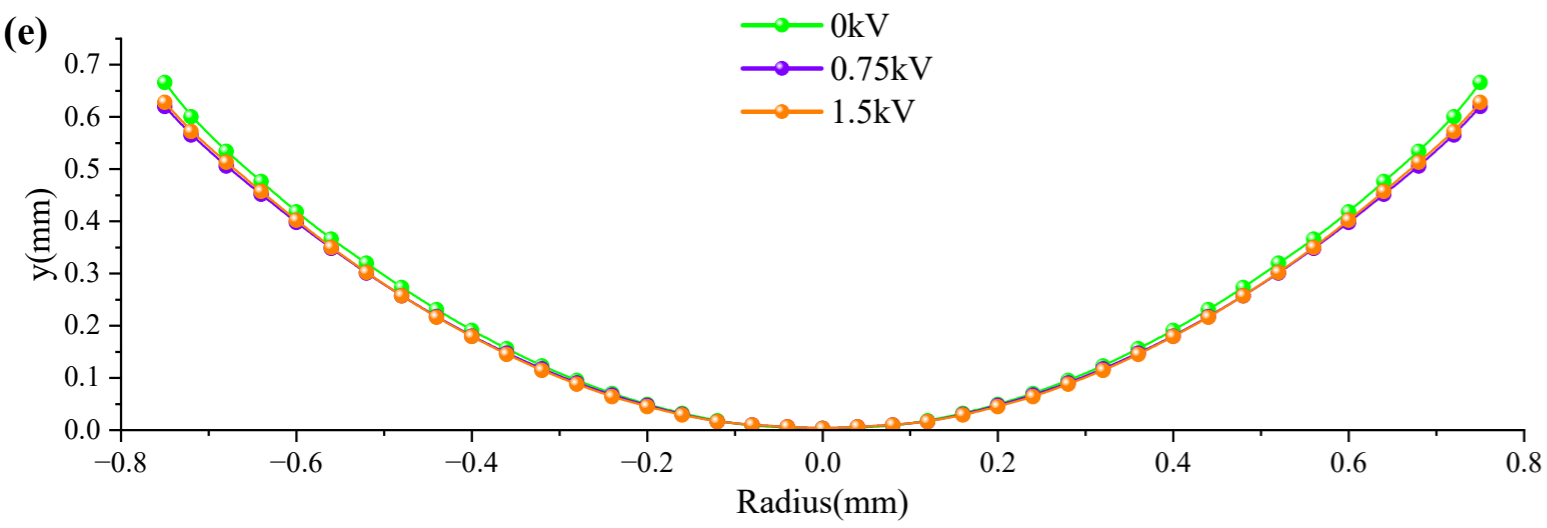

Supplement: Supplementary file 1 [file micromachines-17-00770-s001.zip › figS5.pdf]

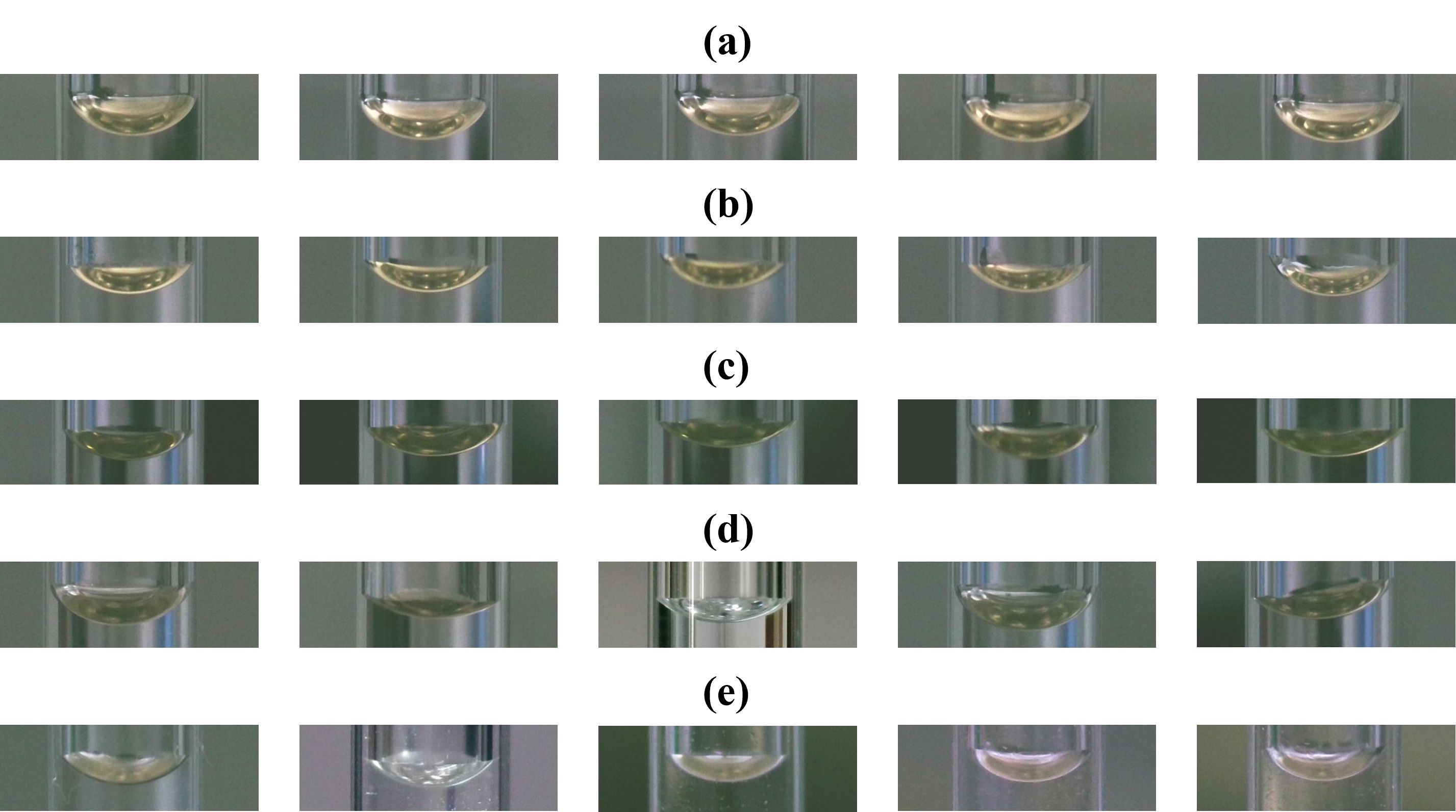

Supplement: Supplementary file 1 [file micromachines-17-00770-s001.zip › figS1.tif]

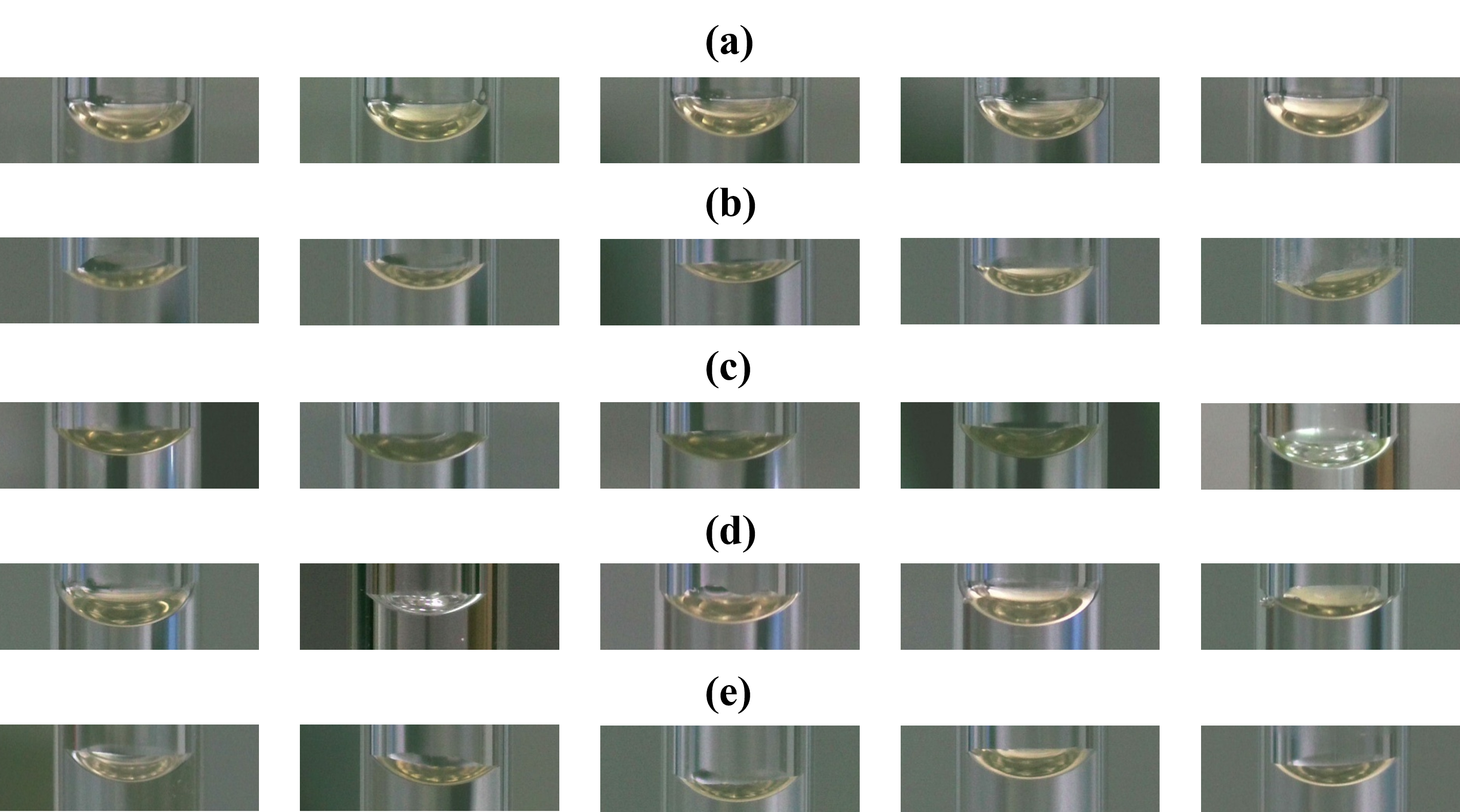

Supplement: Supplementary file 1 [file micromachines-17-00770-s001.zip › figS2.tif]

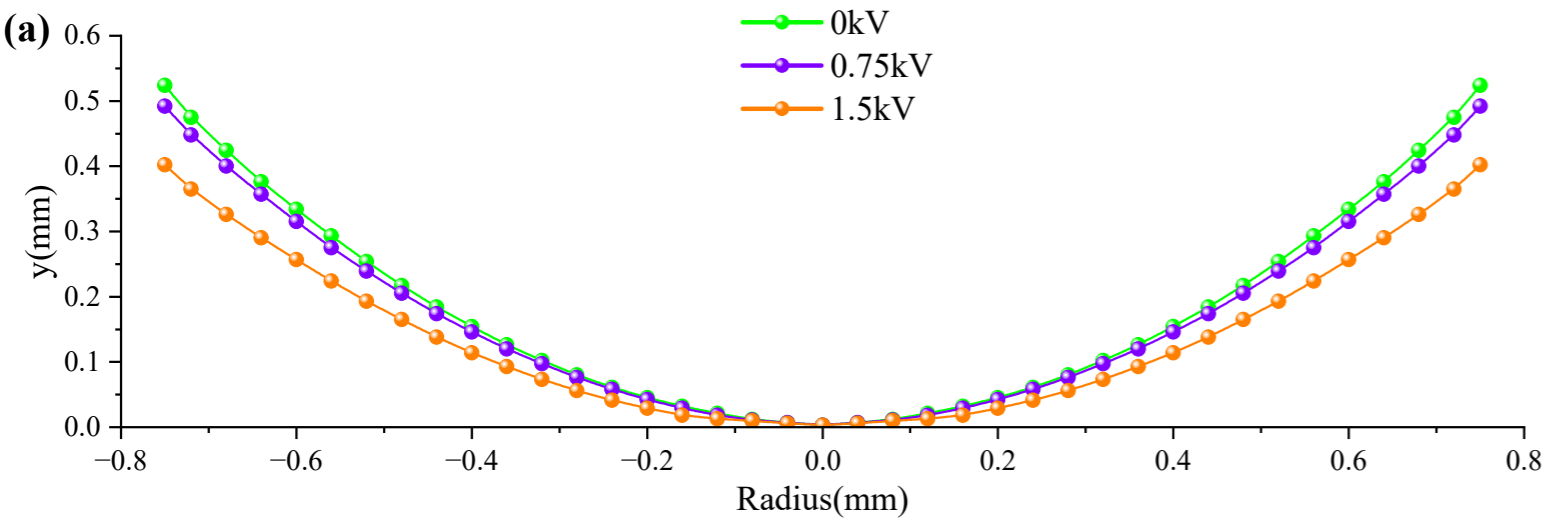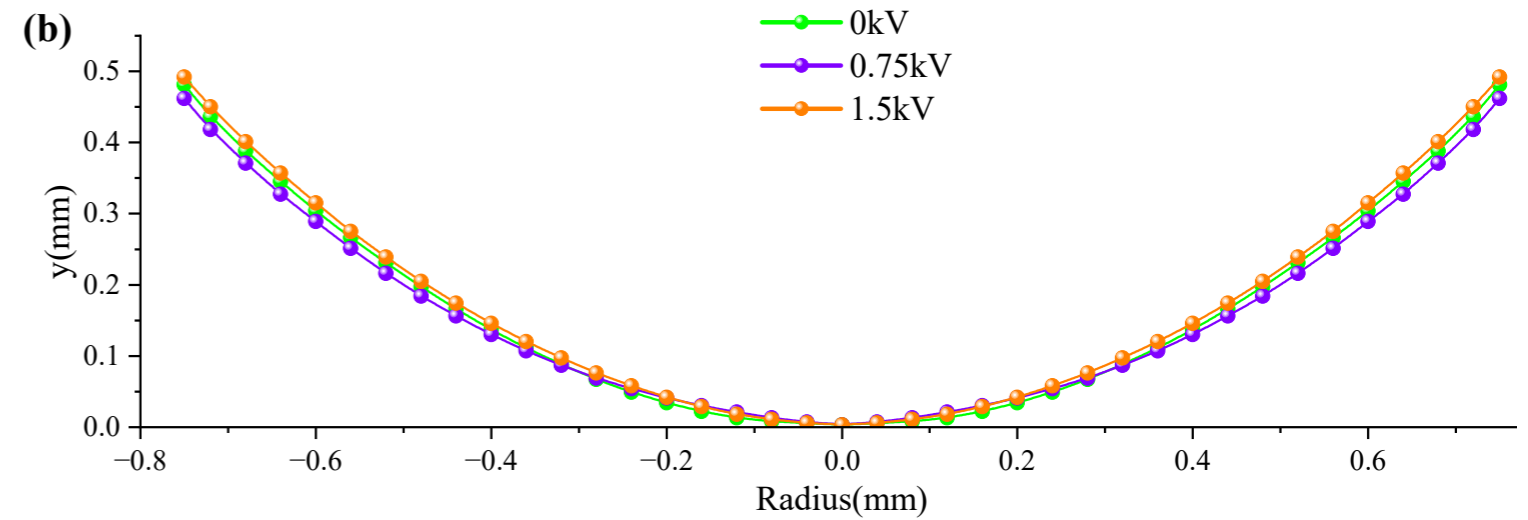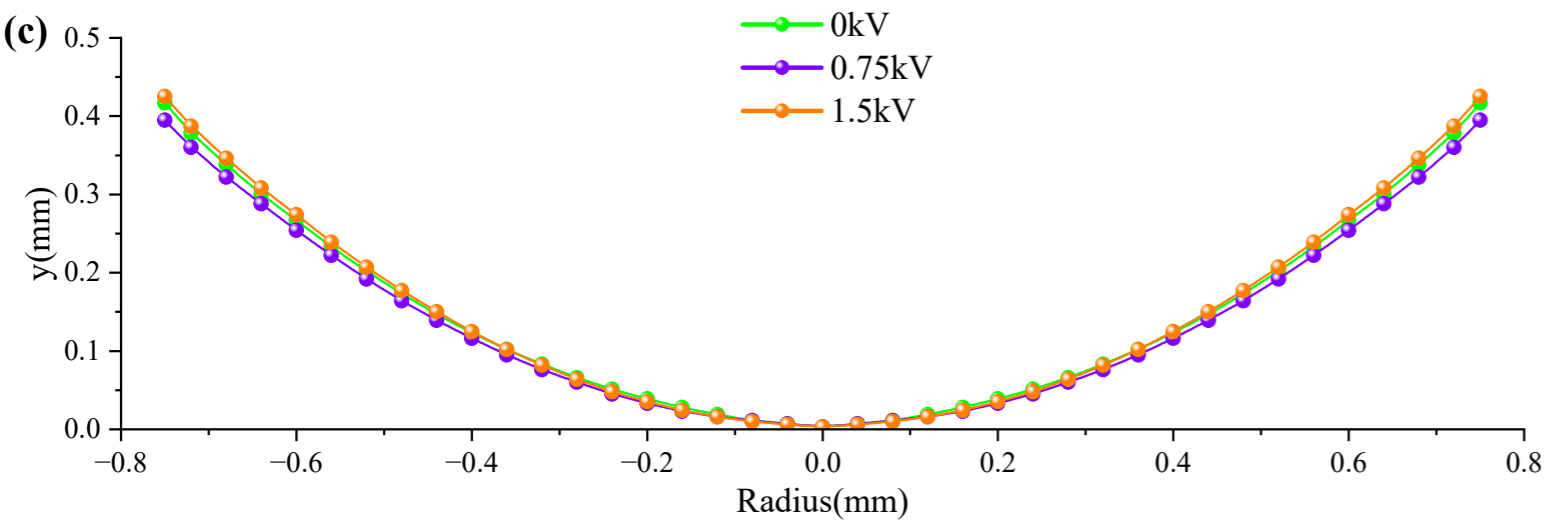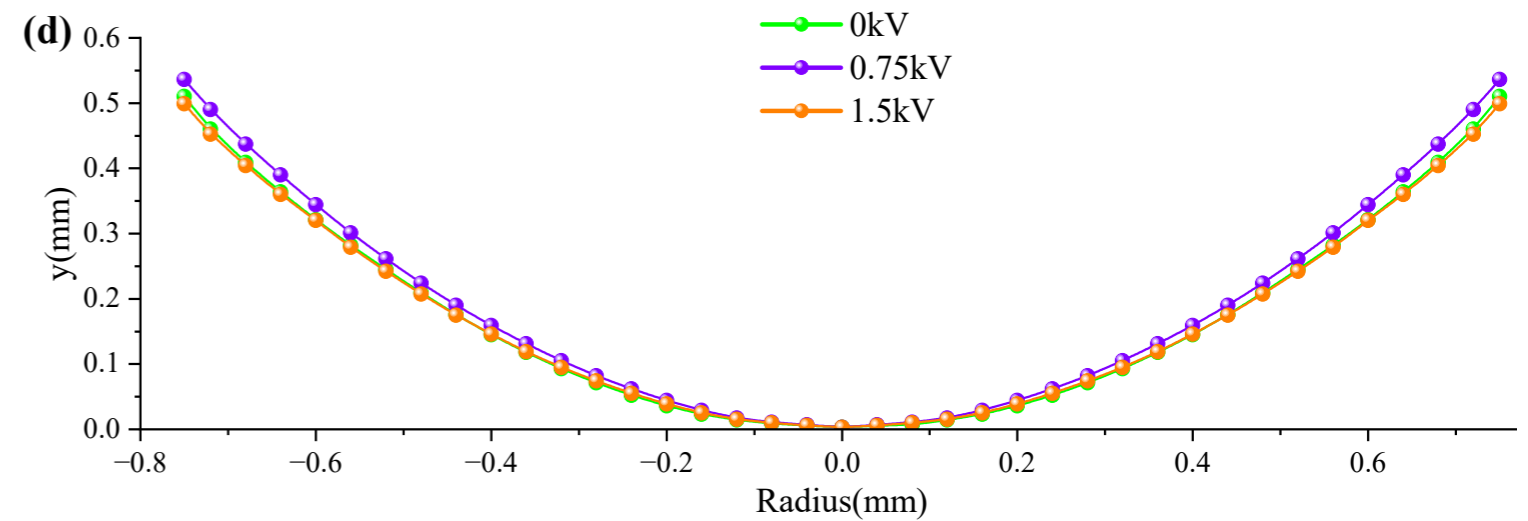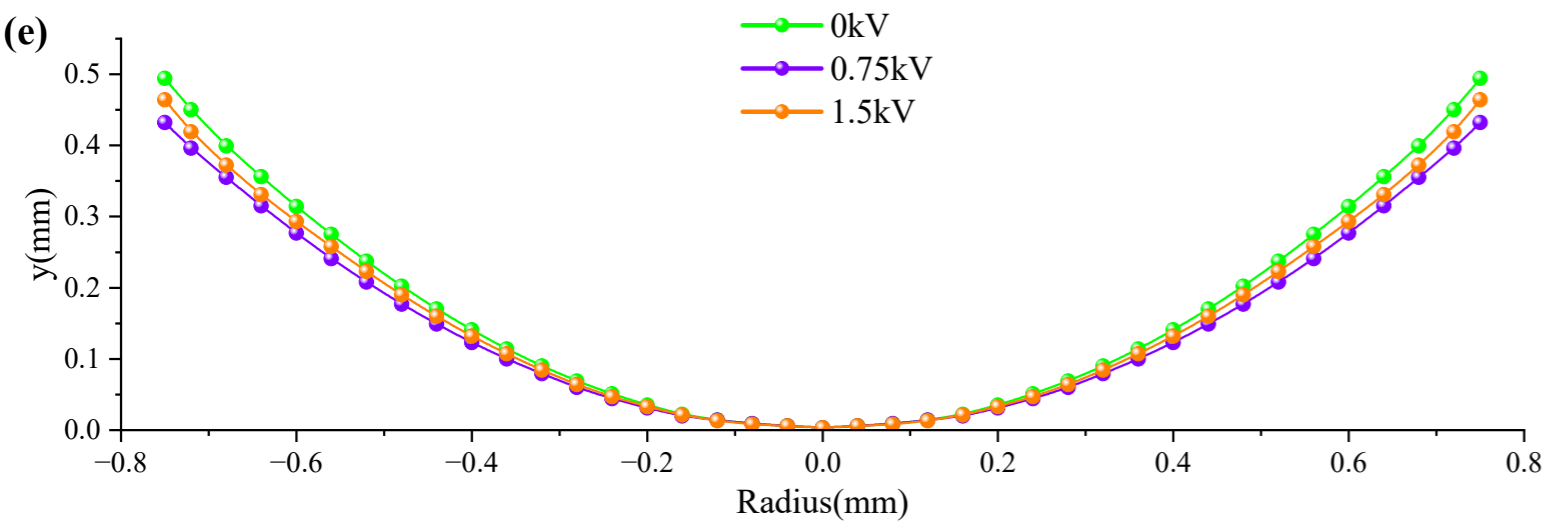

Supplement: Supplementary file 1 [file micromachines-17-00770-s001.zip › figS3.pdf]
